# Salts as Additives: A Route to Improve Performance and Stability of n-Type Organic Electrochemical Transistors

**DOI:** 10.1021/acsmaterialsau.2c00072

**Published:** 2023-03-06

**Authors:** David Ohayon, Lucas Q. Flagg, Andrea Giugni, Shofarul Wustoni, Ruipeng Li, Tania C. Hidalgo Castillo, Abdul-Hamid Emwas, Rajendar Sheelamanthula, Iain McCulloch, Lee J. Richter, Sahika Inal

**Affiliations:** † Organic Bioelectronics Laboratory, Biological and Environmental Science and Engineering Division, King Abdullah University of Science and Technology (KAUST), Thuwal 23955-6900, Saudi Arabia; ‡ Materials Science and Engineering Division, National Institute of Standards and Technology (NIST), Gaithersburg, Maryland 20899, United States; § Department of Physics, Università degli Studi di Milano, Via Celoria 16, I-20133 Milano, Italy; ∥ National Synchrotron Light Source II, Brookhaven National Laboratory, Upton, New York 11973, United States; ⊥ Core Laboratories, KAUST, Thuwal 23955-6900, Saudi Arabia; # Physical Sciences and Engineering Division, KAUST, Thuwal 23955-6900, Saudi Arabia; ¶ Department of Chemistry, Chemistry Research Laboratory, University of Oxford, Oxford OX1 3TA, United Kingdom

**Keywords:** organic electrochemical transistors, electron transporting polymers, aqueous electrolytes, bioelectronics, doping, salt, additive

## Abstract

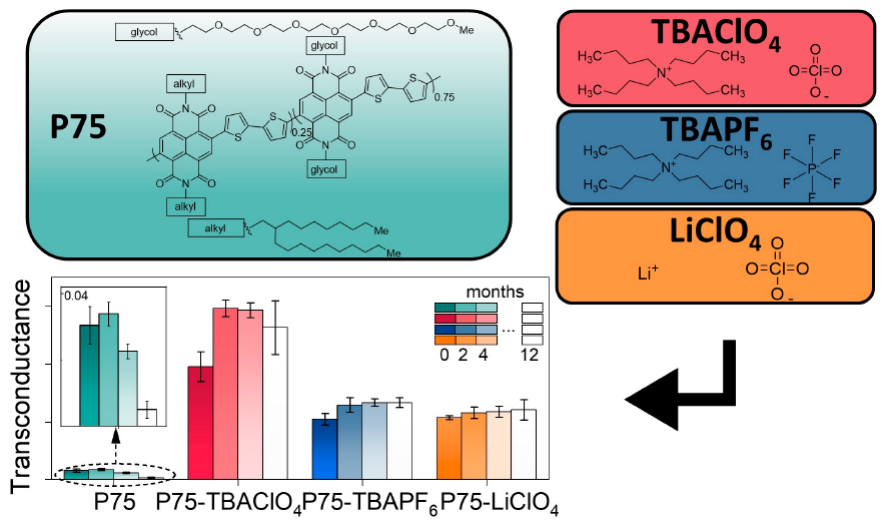

Organic electrochemical transistors (OECTs) are becoming increasingly ubiquitous in various applications at the interface with biological systems. However, their widespread use is hampered by the scarcity of electron-conducting (n-type) backbones and the poor performance and stability of the existing n-OECTs. Here, we introduce organic salts as a solution additive to improve the transduction capability, shelf life, and operational stability of n-OECTs. We demonstrate that the salt-cast devices present a 10-fold increase in transconductance and achieve at least one year-long stability, while the pristine devices degrade within four months of storage. The salt-added films show improved backbone planarity and greater charge delocalization, leading to higher electronic charge carrier mobility. These films show a distinctly porous morphology where the interconnectivity is affected by the salt type, responsible for OECT speed. The salt-based films display limited changes in morphology and show lower water uptake upon electrochemical doping, a possible reason for the improved device cycling stability. Our work provides a new and easy route to improve n-type OECT performance and stability, which can be adapted for other electrochemical devices with n-type films operating at the aqueous electrolyte interface.

## Introduction

The organic electrochemical transistor (OECT) is a transducing amplifier that operates in aqueous electrolytes, therefore, is compatible for use at the interface with biological systems.^1^ The OECT uses an ion-permeable organic semiconductor film in the channel, typically a conjugated polymer, bridging the source and drain electrodes. The electrolyte ions enter the channel because of a field applied through the electrolyte and change the film’s doping state, switching the transistor ON or OFF. Since polymers for OECTs are designed to accommodate ions within their bulk, where they electrostatically compensate for the electronic charges, the transconductance (*g*_m_) of OECTs is intrinsically high—as long as the electronic charges can efficiently move.^2^ A high *g*_m_ translates into high signal-to-noise ratios, useful when OECTs are used to record weak biological signals,^3^ or detect low concentrations of analytes.^4,5^ The fact that the film’s doping state and the duration that it stays in that state can be reversibly controlled with external bias renders distinct conductive states accessible, leveraged to build neuromorphic devices.^6^

Despite the promising application examples of OECTs bearing p-type (hole-transporting) organic semiconductor films, many bioelectronic applications require n-type (electron-transporting) materials with matching performances.^7^ Yet, most n-OECTs demonstrate poorer performance than p-type devices.^8−10^ The main bottlenecks of n-type films are their poor charge carrier mobility, charge localization, and parasitic reactions of the doped film with O_2_.^11,12^ Lowering the lowest unoccupied molecular orbital (LUMO) is usually regarded as a practical approach to improve air stability; this is generally done by lowering the electron-donating strength of the donor units in donor–acceptor-type polymers with the introduction of electron-deficient groups through fluorination,^13,14^ cyanation,^15,16^ and addition of lactone moieties.^17^ Planarization of the backbone through the addition of double bond linkers has been shown to improve charge mobility by reducing the conformational disorder, promoting interchain interactions, and increasing electron delocalization length.^18,19^ Other efforts have focused on tuning the side chain chemistry to improve charge transport. Ethylene glycol (EG) side-chain distance from the backbone and which chemical units they are attached to have been varied to control charge transport and swelling,^20,21^ which were shown to affect OECT performance.^22^ The use of asymmetric branched EG side chains was demonstrated to be a successful alternative to improve the structural order, all while enabling processability from greener (ethanol/water) solvents.^23^ The molecular weight has also influenced intermolecular charge transport, and increasing it led to record-high performances for poly(benzimidazobenzophenanthroline) (BBL).^24^

Postpolymerization routes are easier to apply to improve n-OECT performance as they bypass synthetic procedures. For example, including a “bad” solvent in the n-type polymer-casting solution induced chain aggregation, which led to a morphology enabling better charge transport.^25^ Combining n-type polymers with multiwalled carbon nanotubes (CNTs) facilitated charge transport and improved device speed due to a conductive percolative network provided by CNTs.^26^ An approach rarely explored for OECTs but is very effective for OFETs is using molecular dopants to control materials’ charge carrier density and conductivity.^27^ Dopants, such as cleavable dimeric organometallic complexes,^28^ dimethylaminophenyl dimethylbenzimidazoline (N-DMBI) and dimer derivatives,^29,30^ and tetrakis(dimethylamino) ethylene,^31^ were added to the n-type polymer solutions, and some of them led to films with a conductivity record of 14 S/cm in deoxygenated environments.^30^ Molecular n-doping with such dopants typically occurs via two main mechanisms: Lewis base or redox doping.^27,32^ Current molecular doping strategies have tried to address the main bottlenecks of dopant interfusion into the polymer network and energy levels alignment by using polymer blends to achieve ground-state electron transfer,^33^ enhancing molecular dopant miscibility by tailoring the polarity of polymer side chains^34^ or the dopant,^35^ and matching the energy levels of the neutral and/or anion dopant to achieve double doping.^36^ Alternatively, salt additives, such as the Lewis base tetrabutylammonium fluoride (TBAF)^37^ or alkali metal-based salts,^38^ have been introduced as n-dopants. The doping mechanism of these species is, however, not entirely clarified. A common consensus is that the Lewis acids or bases coordinate with the polymer and induce charge carriers via a redistribution of the electron density^39,40^ or through an anion-assisted charge transfer to the polymer π-electron-deficient unit.^37,41^ We recently showed that chemical doping of an n-type polymer with the Lewis base TBAF improved the n-OECT performance.^42^ However, the solution preparation required a glovebox environment for the transistor coatings due to the air instability of the dopant, which sets a limit for the widespread use of the technique. Second, the mechanism behind the dopant-induced performance improvement has not been well understood. Hence, it is not straighforward to apply the same dopant to other polymers or find dopants that may be more effective.^43^ There is a need for ambient and easy postprocessing methods to improve n-type materials’ performance for bioelectronics applications and to understand the general working mechanism that can be applied to similar materials.

**Figure 1:**
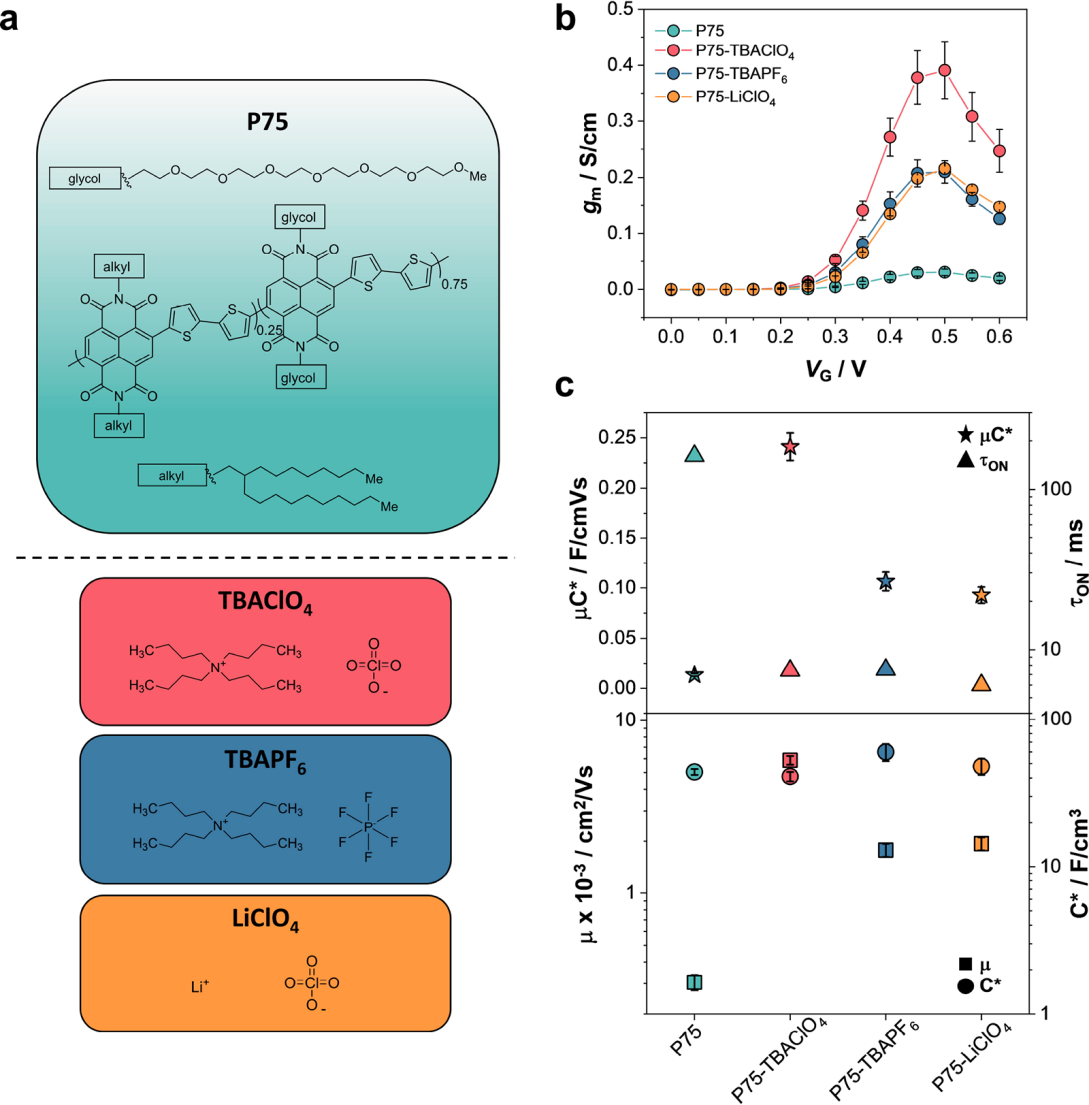
Performance of n-OECTs. (a) Chemical structures of P75 and the salt additives used; (b) *g*_m_ values normalized with respect to film thicknesses of the pristine polymer and the different salt combinations. The *g*_m_ was obtained at *V*_D_ = 0.6 V; (c) μC*, τ_ON_, C*, and μ of each channel material. Error bars represent the standard deviation of at least six different devices.

Here, we introduce organic salts as a solution additive that can be applied in ambient conditions and improves the n-type polymer’s electrochemical characteristics and OECT performance. The n-type conjugated polymer we chose is a copolymer based on a naphthalene-1,4,5,8-tetracarboxylic diimide (NDI) coupled with an unsubstituted bithiophene (T2) backbone from now on called P75 (Figure 1a). The side chains on the NDI unit are branched alkyl or linear EG groups mixed in the copolymer with a monomer ratio of 25:75, respectively. As salts, we chose tetrabutylammonium perchlorate (TBAClO_4_), tetrabutylammonium hexafluorophosphate (TBAPF_6_), and lithium perchlorate (LiClO_4_), salts that are typically used for the electropolymerization of conducting polymers^44,45^ and in studies that investigate the influence of electrolyte species on electrochemical device performance.^46^ The OECTs with channels cast from salt-bearing solutions showed a 10-fold increase in *g*_m_ and faster operation. These devices retained their initial performance metrics over a year of storage, whereas the pristine P75 OECTs demonstrated continuous degradation. The salt-cast films exhibited much higher electron mobilities yet similar volumetric capacitances. Electron paramagnetic resonance (EPR) spectroscopy found only weak intrinsic doping by salts. Raman and Fourier transformed infrared (FTIR) spectroscopies evidenced interactions of salt additives with the T2 unit and C=O group of the NDI and improved backbone planarity and charge delocalization due to these interactions. The salt-cast films showed a subtle contraction of the π-lattice revealed through grazing incidence wide-angle scattering (GIWAXS). The film surface was, however, significantly more porous when cast from salt-bearing solutions. Using X-ray photoelectron spectroscopy (XPS) and secondary ion mass spectrometry (SIMS), we found only the salt cations inside the films and only in as-cast conditions, suggesting that although the ions diffuse out of the films after electrolyte exposure, their effect on film structure is permanent. Our work suggests organic salts added to the polymer solutions in ambient conditions as an easy route to address the main bottlenecks of n-OECTs, i.e., performance and stability, and constitutes a simple approach without relying on chemical innovation.

## Material and Methods

### Materials

An NDI-T2-based copolymer, P-75, was synthesized according to a previously reported protocol, with one monomer bearing alkyl side chains, and the second unit linear ethylene glycol side chains, in a 25:75 ratio, respectively.^47^ Tetrabutylammonium perchlorate (TBAClO_4_), tetrabutylammonium hexafluorophosphate (TBAPF_6_), and lithium perchlorate (LiClO_4_) were purchased from Sigma-Aldrich and used as received.

### OECT Fabrication

OECTs were microfabricated on glass substrates based on established protocols using standard photolithography and Parylene-C peel-off techniques. P-75 solutions were prepared at a concentration of 4 mg/mL in chloroform. Salt additives were introduced at 20% molar concentration in chloroform. Solutions were heated at 45 °C for 1 h and then spin-coated on devices at 1000 rpm (1 rpm = 2π/60 rad/s) for 30 s, with an acceleration speed of 500 rpm/s. All devices had the same channel (*W* = 100 μm, *L* = 10 μm) and gate (500 × 500 μm) dimensions. Output characteristics were acquired at a scan rate of 0.1 V/s in both directions (forward and backward scans) until the transistors reached stable operation.

Devices were stored in vacuum for shelf life stability evaluation. They were immersed in 0.1 M NaCl for 20–30 min before the measurements to allow the films to swell and achieve stable operation faster. OECTs were rinsed thoroughly with deionized water in between days of measurements and dried with N_2_.

### Electrochemical Characterization

Cyclic voltammograms were recorded with a Metrohm Autolab potentiostat using a standard three-electrode setup, 0.1 M NaCl as the electrolyte, and a scan rate of 50 mV/s in air or an N_2_-filled glovebox. The polymers were coated on gold-sputtered glass wafers (2 × 2 cm) according to the procedure described above, addressed as the working electrode, with a platinum wire as the counter electrode and an Ag/AgCl as the reference electrode. Volumetric capacitance (C*) values were extracted using electrochemical impedance spectroscopy (EIS), performed on microelectrode arrays made of Au electrodes coated with the polymers to span a range of different sizes (from 9 × 10^–4^ to 0.01 cm^2^). A Metrohm Autolab potentiostat applied an electrochemical doping potential of −0.5 V vs Ag/AgCl with a 10 mV modulation amplitude and a frequency (*f*) range from 100 kHz to 0.1 Hz. Capacitance (*C*) was calculated from the imaginary component of impedance (*Z*^img^) using the following equation:
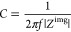
1where C* was obtained from the slope of the extracted *C* versus film volume curve.

### UV–Vis–NIR Absorption and Energy levels

UV–vis–NIR absorption spectra were obtained using a Carry 5000 UV–vis–NIR spectrometer (λ_max_ = 3300 nm). Photoelectron spectroscopy in air (PESA) measurements were performed with a Riken Keiki AC-2 PESA spectrometer. Samples for PESA were prepared on glass substrates by spin-coating polymers with the procedure described above. Note that we assumed that the ionization potential (IP), measured by PESA, was similar to the highest occupied molecular orbital (HOMO) of the polymers. The optical band gap of each polymer was extracted from the onset of the absorption spectra. The electron affinity (EA) was deduced from the IP and band gap values. Similarly, we assumed that the EA was comparable to the LUMO of the polymer.

### Electron Paramagnetic Resonance

X-band Bruker CW-EMX PLUS EPR spectrometer equipped with a super high Q (ER 4122 SHQ) resonator was used to record EPR spectra at 25 dB microwave attenuation and 100 kHz and 5 GHz modulation frequency and amplitude, respectively. Bruker Xenon software (Bruker BioSpin, Rheinstetten, Germany) was used for data collection and analysis.

### X-ray Photoelectron Spectroscopy

XPS measurements were performed using a Kratos Axis Supra instrument equipped with a monochromatic Al Kα X-ray source (*h*ν = 1486.6 eV) which was operated at a power of 150 W and under UHV (in the range of ∼10^–9^ mbar). All spectra were recorded in hybrid mode using electrostatic and magnetic lenses. The survey and high-resolution spectra were acquired at fixed S-5 analyzer pass energies of 80 and 20 eV, respectively. The obtained spectra were calibrated using the reference C 1s at 284.8 eV. The spectra were deconvoluted using XPSPeak4 software with Gaussian and Lorentzian methods, while the background was subtracted using the linear method.

### Atomic Force Microscopy (AFM)

AFM measurements were performed with a Veeco Dimension 3100 scanning probe system. Samples were prepared on Au-coated glass wafers according to the coating procedure described above. Dry film experiments were performed using FESPA-V2 probes commercialized by Bruker (nominal resonant frequency: 75 kHz, spring constant: 2.8 N/m). Wet film scans, with 0.1 M NaCl as the electrolyte, were obtained using the Bruker tapping mode in a fluid module mounted with Scanasyst-fluid probes commercialized by Bruker (nominal resonant frequency: 150 kHz, spring constant: 0.7 N/m). The sample and the probe were immersed in 0.1 M NaCl buffer electrolyte while scanning. All images were acquired on a 2 μm × 2 μm surface map at 0.5 Hz with a resolution of 512 pixels/line. First, AFM measurements were performed with dry *as-cast* films, which were first exposed to the electrolyte and then electrochemically doped. “Dry morphology after doping” images were acquired by rinsing the samples with deionized water and then leaving them to dry in ambient conditions for at least a day before the measurements. Gwyddion software was used for statistical data and post-treatment.

### Grazing Incidence X-ray Scattering

Films were prepared as described above on 1 cm^2^ p^++^ silicon substrates. The data were collected at Brookhaven National Lab, National Synchrotron Light Source II (NSLS-II), beamline 11-BM Complex Materials Scattering. The incident X-ray energy was 13.5 keV. For wide-angle scattering, a detector distance of 258 mm and an incident angle of 0.14° with respect to the substrate were used. For small-angle scattering, a detector distance of 2 m was used. The detector calibration was performed using a silver behenate crystal. Scattering data were processed using the Nika software package for Wavemetrics Igor Pro.^48^

### Electrochemical Quartz Microbalance with Dissipation Monitoring (e-QCMD)

QCM-D measurements were conducted using a Q-sense analyzer (QE401, Biolin Scientific AB) with Cr/Au-coated quartz crystals before (used as reference) and after coating with the polymer films in the degassed electrolyte to prevent any effect of ORR on the current values. First, the bare (noncoated) sensors were measured in air, followed by the introduction of degassed buffer (0.1 M NaCl) at 100 μL/min to ensure accurate swelling calculations, accounting for the change in media density in the QCM-D chamber. Next, the sensors were coated with the polymers following the procedure described above. The frequency (Δ*f*) and the dissipation (Δ*d*) signals of the quartz-coated sensors were first measured in air, then in degassed NaCl 0.1 M buffer flown at a speed of 100 μL/min. After stabilization of the signals (Δ*f* < 0.1 Hz per 5 min), the films were biased using an Autolab PGstat128N potentiostat coupled to the QSense electrochemistry module (QEM 401). The module encompassed a three-electrode configuration with an Ag/AgCl reference electrode, a Pt counter electrode, and the polymer-coated Au QSensor acting as the working electrode with an active electrochemical area of 0.7854 cm^2^. Three consecutive pulses of −0.5 V vs Ag/AgCl and +0.3 V vs Ag/AgCl were applied, each for 1 min. We used the fifth, seventh, and ninth harmonics for data analysis. The measured shifts in the frequency of the sensors were converted into changes in mass (Δ*m*) using the Sauerbrey equation:
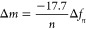
2where *n* is the number of the overtone selected for the mass quantification and −17.7 is a constant determined by the crystal’s resonant frequency, active area, density, and shear modulus. The validity of the Sauerbrey equation for our analysis is guaranteed since Δ*d*/Δf was smaller than 4 × 10^–7^, i.e., the characteristic threshold to consider a layer rigid and safely apply the Sauerbrey relation.^49^ We used the QSoft software function “stitch data” to calculate the respective films’ areal mass in dry and swollen states. The swelling was calculated as the percentage change in volume relative to the dry volume:
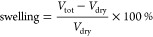
3Na^+^ density in the films upon doping was calculated through the number of electrons generated in the polymer film recorded as the reduction current and assuming a 1:1 electron-to-cation coupling. H_2_O density was then extracted by subtracting the injected Na^+^ mass (3.81763 × 10^–23^ g/atom) from the total mass recorded by the eQCM-D and dividing the result by the molecular mass of water (2.99 × 10^–23^ g/molecule). Possible contributions of counterions or faradaic reactions on mass uptake and measured current were not considered.

### Raman Spectroscopy

A Cr/Au (10/100 nm) layer was sputtered on a cleaned glass wafer. The different polymer solutions were then spin-coated on 1 cm x 1 cm substrates following the same coating procedure described above. All films had a similar thickness. Raman spectra of all dry polymer thin films in *as-cast* and after immersion in 0.1 M NaCl electrolyte (*exposed*) were collected using a WITec Apyron Raman spectrometer equipped with a 633 nm laser and 100× Zeiss lens, NA = 0.9, and a power level of 100 μW to avoid photothermal effects. Spectra were acquired using the stitching mode of the WITec Five software between 800 and 2000 cm^–1^, with a dispersion grating of 1800 g/mm to ensure high-resolution acquisition. For each sample, we kept an integration time of 2 s and averaged the spectra over 30 accumulations to define the representative spectrum. Raman spectra were also collected at multiple positions to validate the samples’ chemical and optical homogeneity. The Raman spectra were obtained by removing the baseline using the WITec Five software feature and after normalization to the Я-mode peak intensity of the C=N units (1574 cm^–1^).^50,51^ The spectral resolution arises statistically from the mathematical analysis. Given the small size of fiber core diameter, we estimated it to be ∼0.1 cm^–1^.

### Fourier Transform Infrared Spectroscopy

FTIR spectra were recorded using a Thermo Scientific Nicolet iS10, between 500 and 4000 cm^–1^, at room temperature in attenuated total reflection (ATR) mode. A single spectrum was obtained from 64 averaged scans, while OMNIC FTIR software was used to correct the baseline.

## Results and Discussion

The salts, i.e., TBAClO_4_, TBAPF_6_, and LiClO_4_, were introduced at 20% molar concentration in each polymer solution (see Figure 1a for the chemical structures). All polymer solutions were spin-coated on microfabricated OECTs with identical channel dimensions. These devices were characterized in an aqueous electrolyte (0.1 M NaCl) using an Ag/AgCl gate electrode. The steady-state performance of an OECT is characterized by its *g*_m_, which is governed by the channel material’s electronic charge mobility (μ) and volumetric capacitance (C*) according to the following equation derived for current saturation conditions:^52^
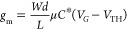
4where *W*, *d*, and *L* represent the width, thickness, and length of the channel, respectively, *V*_G_ is the gate voltage, and *V*_TH_ is the threshold voltage. Figure 1b shows that all of the salt additives-based OECTs perform much better than the pristine device, displaying 1 order of magnitude improvement in the *g*_m_ (see Figure S1 for the output and transfer curves). Note that devices made from solutions with higher salt concentrations (40%) did not switch ON in this biasing range. All devices show a low level of hysteresis (Figure S1). Among the different salt additives tested, the P75-TBAClO_4_ combination has the best performance, with a normalized *g*_m_ about two times higher than the two other salt combinations and 20 times higher than the pristine film. The off currents measured at *V*_G_ = 0 V are similar for all salt-treated polymers, and the on–off ratio is higher than the pristine device, as high as 2 orders of magnitude for P75-TBAClO_4_ (Table 1).

**Table 1:** OECT Performance Characteristics of P75, P75-TBAClO_4_, P75-TBAPF_6_, and P75-LiClO_4_

polymer	*g*_m_^a^ (S/cm)	*V*_TH_^b^ (V)	C*^c^ (F/cm^3^)	μ^d^ (cm^2^/V s)	μC*^e^ (F/cmVs)	*I*^on^/*I*^off^^f^	τ_ON_|τ_OFF_^g^ (ms)
P75	0.03 ± 0.01	0.28 ± 0.01	44 ± 2	3.05 × 10^–4^ ± 3.16 × 10^–5^	0.01 ± 0.01	10^2^	161.6|144.1
P75-TBAClO_4_	0.39 ± 0.05	0.29 ± 0.01	41 ± 3	5.88 × 10^–3^ ± 3.34 × 10^–4^	0.24 ± 0.01	10^4^	7.4|15.6
P75-TBAPF_6_	0.21 ± 0.02	0.29 ± 0.01	60 ± 8	1.78 × 10^–3^ ± 1.55 × 10^–4^	0.11 ± 0.01	10^3^	7.5|18.5
P75-LiClO_4_	0.22 ± 0.01	0.30 ± 0.01	48 ± 6	1.93 × 10^–3^ ± 1.69 × 10^–4^	0.09 ± 0.01	10^3^	6.0|19.9

a Obtained from normalizing the maximum *g*_m_ by the average thickness for each polymer film.

b The *X*-intercept of the maximum slope of the linear portion of √*I*_D_ vs *V*_G_ curve.

c Calculated from the eq 1 at *f* = 1 Hz.

d Estimated by dividing μC* with C*.

e Estimated from the slope of *g*_m_ vs (*Wd*/*L*) × (*V*_G_ – V_TH_) using eq 4.

f Obtained by calculating *I*_D_ (*V*_G_ = 0.6 V)/*I*_D_ (*V*_G_ = 0 V) at *V*_D_ = 0.6 V.

g Calculated from a monoexponential decay fit of the channel transient response to a square *V*_G_ pulse (Figure S4). The values in the table represent the average ± standard deviation obtained from at least six different devices.

The *V*_TH_ does not change significantly upon salt introduction (Table 1), suggesting little alterations to the energetic levels of the polymer film, confirmed with UV–vis–NIR absorption spectroscopy and PESA measurements (Figure S2 and Table S1). In the UV–vis–NIR spectra of thin films, we do not observe any particular difference between the polymers, displaying the two characteristic absorption features of NDI-T2-based polymers, i.e., a π–π transition at around 400 nm and internal charge transfer (ICT) feature around 720 nm. We also do not detect polaron/bipolaron signals that would arise from chemical or electrochemical doping upon salt addition. However, for the P75-TBAClO_4_ sample, we see a 2 nm blue shift of the π–π transition around 400 nm compared to the pristine P75 film, as well as the appearance of a slight energy shoulder around 900 nm (Figure S2). Further, only a slight red shift (∼1 nm) of the ICT feature is visible upon salts introduction (Figure S2), suggesting little differences in the packing arrangement of the polymer chains. We characterized the mixed conduction figures of merits (μ and C*) for each film. Figure 1c and Table 1 show that μ increases a minimum of 5 times with TBAPF_6_ addition and a maximum of an order of magnitude with TBAClO_4_ addition. On the other hand, the C* value only slightly increased, from 44 to a maximum of 60 F/cm^3^ (see Figure S3 for *C* versus volume plots). The μC* is thus enhanced for salt-based materials, with the highest magnitude reached for P75-TBAClO_4_. Moreover, we measured the transient response of each OECT. We found significantly faster switch ON speeds (τ_ON_) for the salt-based channels (Figures 1c and S4), in line with the improved charge mobility.^53^

**Figure 2:**
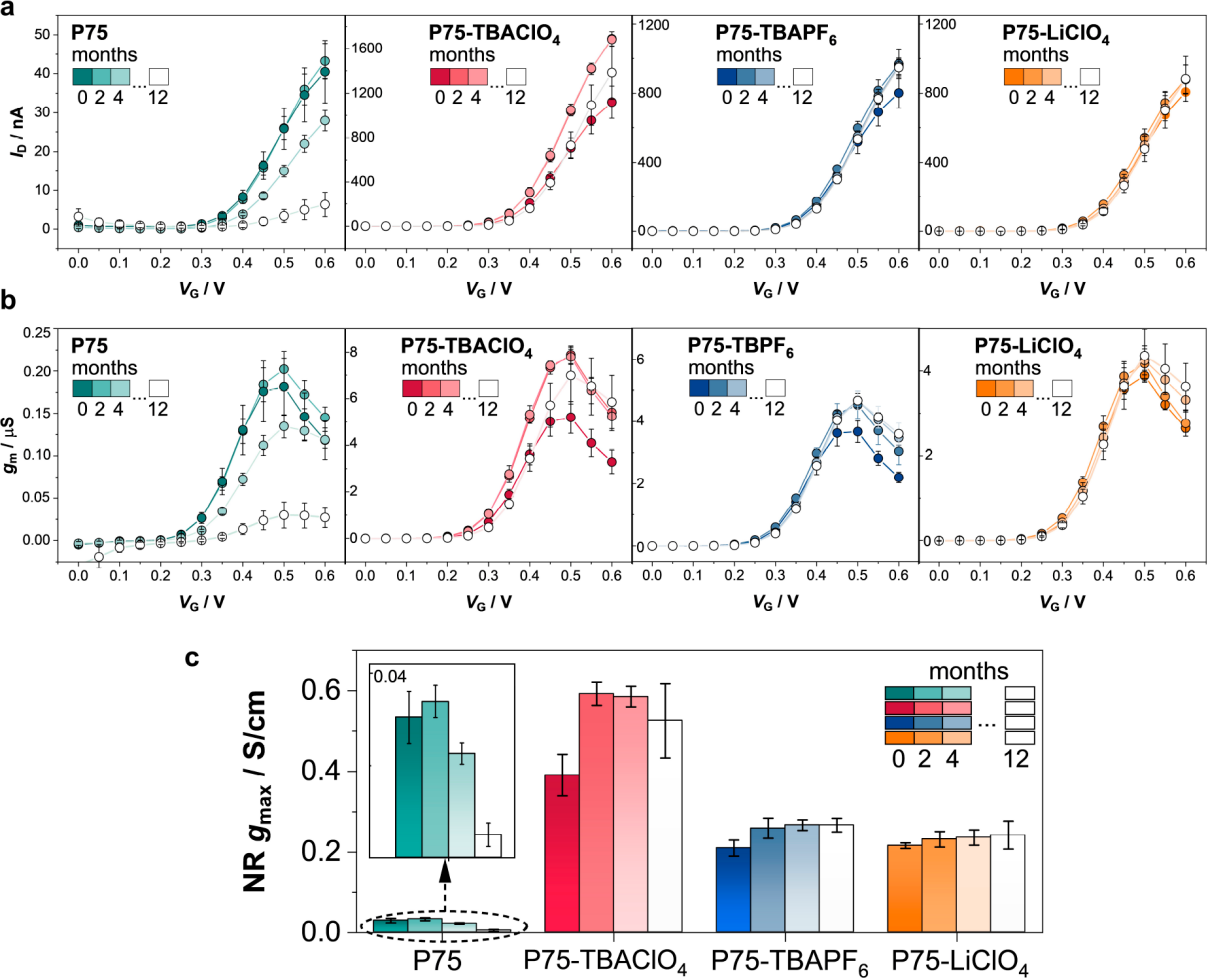
Shelf life stability of P75, P75-TBAClO_4_, P75-TBAPF_6_, and P75-LiClO_4_. (a) Transfer curves and (b) *g*_m_ vs *V*_G_ plots of the devices cast from various salt formulations. The devices were measured after deposition (0 months) and after 2, 4, and 12 months of storage in a vacuum. The scan rate was 0.1 V/s. (c) Normalized *g*_m_ (with respect to channel thickness) after deposition (0 months), 2, 4, and 12 months of storage. Error bars represent the standard deviation measured from at least six different devices.

Not only the steady-state performance but also the shelf life and operational stability of these devices improved upon salt introduction. Figure 2a shows that, after 4 months of storage, the pristine P75 OECTs exhibit a drastic drop in their performance (by almost 50%), while the salt-based devices operate without degradation. After a year of storage, pristine devices continue to degrade and show more than an 80% decrease in *g*_m,_ while salt-based devices maintain their initial performance (Figure 2b,c). Besides shelf life stability, salts also improve operational stability. When stressing the device with short ON and OFF voltage pulses at the gate electrode for over 2 h, all devices show a slight decrease of the drain current (*I*_D_) at the beginning of the biasing; however, salt-based devices recover over time, while the pristine one displays a 60% *I*_D_ loss at the end of the cycle (Figure S5).

Introducing salt additives in the polymer solution enhances the steady-state performance of n-OECTs and their stability. Although our observations so far, e.g., similar UV–vis–NIR spectra, unchanged energetics as well as similar OFF currents and capacitances, do not hint chemical doping as the source of the performance improvement, electron paramagnetic resonance spectrum shows a small signal arising from unpaired electrons in *as-cast* salt-based thin films, which is not present in the pristine polymer (Figure S6). However, the EPR signals are low and relatively noisy. Therefore, we anticipate that even if salts generate unpaired electrons, these cannot be alone responsible for the significant changes we see in device performance.

n-Type polymers have been shown to perform oxygen reduction reaction (ORR) under reductive potentials in aqueous media.^54^ One possible reason for performance improvement might be that the adverse effects of ORR, which interfere with charge generation and transport, may be circumvented in the presence of salts in the film. We thus probed the influence of O_2_ on device performance by recording device characteristics in a N_2_-saturated glovebox (Figure S7). We observe that O_2_ does not dramatically influence the OECT characteristics, with a maximum of 15% increase in the *I*_D_ under inert conditions for all devices. However, given that the OECTs were characterized using a nonpolarizable gate electrode (Ag/AgCl), we expect the gate electrode to supply additional charge carriers to maintain the applied gate potential in the event of ORR. The higher gate currents observed in ambient conditions indeed confirm ORR, but the magnitude of these currents is also the same for all devices (Figure S8). We also investigated the extent of ORR in the films using cyclic voltammetry (CV) performed in air/ambient conditions and a N_2_-saturated glovebox using a degassed electrolyte (Figure S9). We observe a general downshift of the CV curves when measured in air and that the currents are similar for the overall voltammogram in both atmospheres. In ambient conditions, a first reduction peak appears around −0.3 V, which is absent in N_2_ conditions, attributed to ORR.^55^ In N_2_ conditions, we observe two reduction and oxidation couples for the salt-bearing P-75 films, attributed to the electrochemical doping and dedoping of the NDI with Na^+^ cations, respectively. These results suggest that the salt additives do not prevent ORR inherent to the P75 film. Thus, the origin behind the increase in device performance should be sought elsewhere, possibly, in film microstructure and morphology that may change if the chains interact with salt.

**Figure 3:**
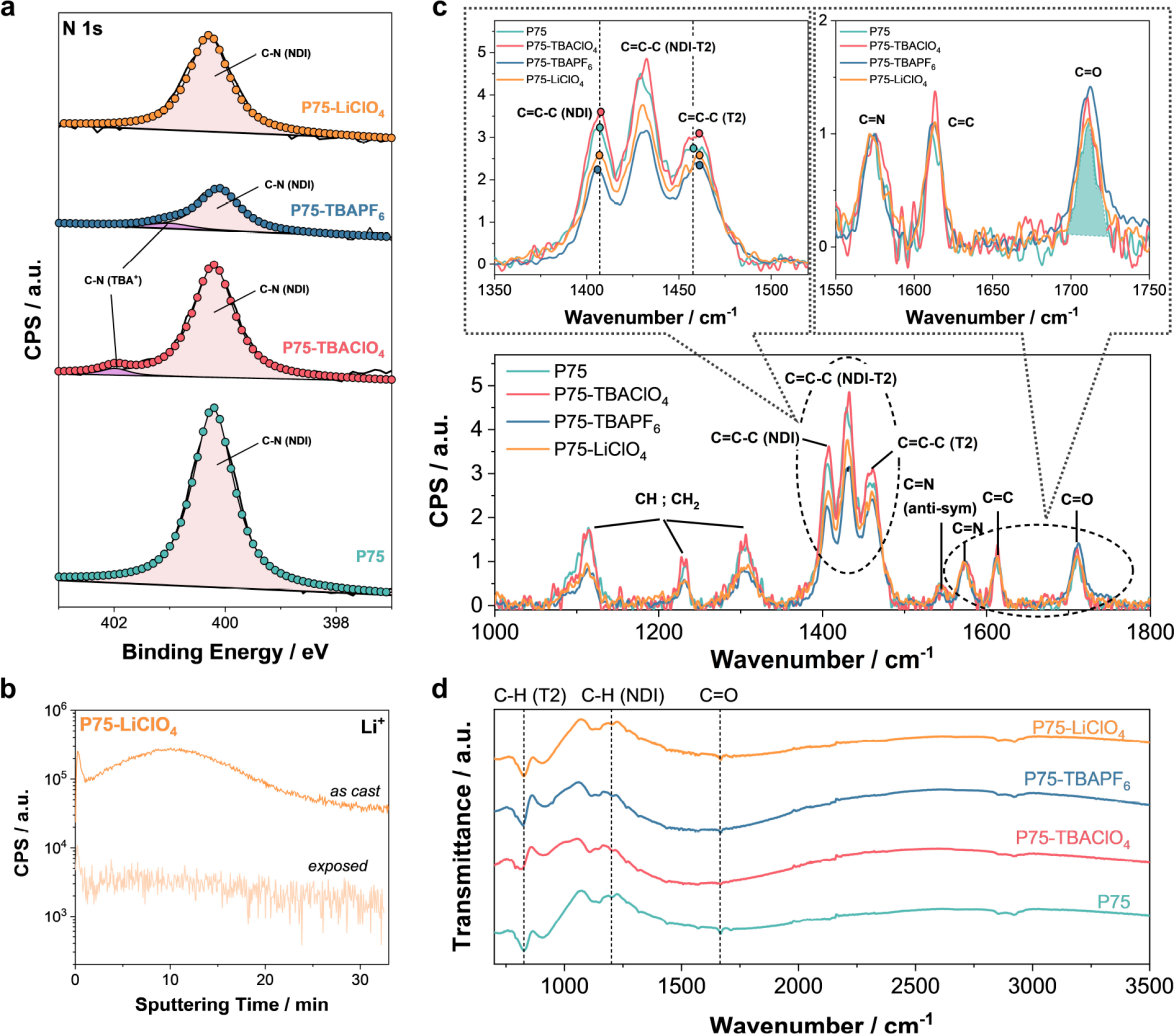
Tracking the salts inside polymer films. (a) High-resolution XPS spectra of N 1s spectra of P75, P75-TBAClO_4_, P75-TBAPF_6_, and P75-LiClO_4_*as-cast* films. (b) Secondary ion mass spectrometry of Li^+^ signal in *as-cast* and *exposed* (electrolyte exposure to 0.1 M NaCl for 1 h) thin film of P75-LiClO_4_. (c) Raman spectra of P75, P75-TBAClO_4_, P75-TBAPF_6_, and P75-LiClO_4_*as-cast* films. Insets show a zoom-in of the NDI-triplet (left) and the C=O broadening (right). The dotted line on the left inset, centered on the C=C–C (T2) peak, serves as a guide to the eye to illustrate the shift in peak position with salts addition, where the peak position is represented by the circle of corresponding colors. In the inset on the right-hand side, the C=O peak area of the P75 is highlighted to illustrate more clearly the broadening of the peak upon salt introduction. (d) FTIR spectra of P75, P75-TBAClO_4_, P75-TBAPF_6_, and P75-LiClO_4_*as-cast* films.

To probe salt-polymer interactions, we first tracked whether the salts reside in the films and where they locate. We used X-ray photoelectron spectroscopy, which probes the film surface, and secondary ion mass spectrometry (SIMS), which allows us to access the elemental distribution through the bulk. In the XPS spectra of as-cast films from the TBA-based formulations, we detect the N 1s peak in (401.99 eV), suggesting the presence of the TBA^+^ cation in the films (Figure 3a). We could not reliably probe the Li cation in the LiClO_4_-based film, as the Li^+^ peak overlapped with the satellite peak of Au 5p_3/2_ (Figure S10). None of the anion-representative elements (Cl, P, F) could be found in the corresponding spectra (Figure S11), suggesting either that the anions are not present in the film or that their concentration is below the machine detection limit. Since in the OECT configuration the films will be exposed to an aqueous electrolyte, we investigated the XPS spectra of thin films after they were immersed in the electrolyte, 0.1 M NaCl solution, for 1 h. Once the films were immersed in the electrolyte, their spectra no longer showed signals from TBA^+^ (Figure S12).

We could not observe any signs of the other components of the salt additives, suggesting that the salt species in the *as-cast* films migrated out of the films once exposed to the electrolyte. To validate these findings, we investigated the elemental distribution profile of our films with SIMS. We could not differentiate the TBA^+^ cation signal from the NDI signal in SIMS. However, SIMS profiles confirm the presence of Li^+^ in P75-LiClO_4_ in the as-cast form and a net decrease in its signal intensity after the film was *exposed* to the electrolyte (Figure 3b). The profiles of negative elements (see Figure S13 for F-, P-, and Cl-specific spectra) show the presence of F^–^ in both *as-cast* and *exposed* P75-TBAPF_6_, while no P^–^ signal is observable in any of the conditions. We detect Cl^–^ in all samples, displaying a decrease in content after electrolyte exposure. However, given that P75-LiClO_4_ and P75, P75-TBAPF_6_*as-cast* films display a Cl^–^ signal with the same intensity, this signal is very likely due to interference/contamination.

These results suggest that salt species interact with the polymer chains, accommodating in the films during casting, and migrate out once interfacing with the electrolyte. Pearson’s HSAB theory describes interactions between a solvent (here, chloroform) and the anions/cations of a salt, providing a quantitative description of their Lewis acidity and basicity.^56,57^ Anions and cations can exist in a solution in various ways, affecting how they interact with other species. They can be covered by a solvent shell to form contact ion pairs (poor solvation), be completely solvated and separated in the form of ion–solvent pairs, or either one of them can be preferentially solvated by the solvent.^58^ These interactions are dictated by the donor and acceptor numbers of the species, DN and AN, respectively. The DN of chloroform (4.0) is much smaller than the DN of any of our anions (DN(ClO_4_^–^) = 35.31 and DN(PF_6_^–^) = 10.46), suggesting that the cations, TBA^+^ and Li^+^, are less likely to be solvated by chloroform and more likely to exist as ion pairs or naked.^56,58^ The acid/base strength combination is also an important parameter that can influence the ion’s state and how tightly they are bound to each other. The Pearson HSAB theory states that hard acids pair with hard bases, while softer acids prefer softer bases. Here, ClO_4_^–^ can be considered a hard Lewis base, while PF_6_^–^ is regarded as a soft Lewis base;^58,59^ for the cations, Li^+^ and TBA^+^ are characterized as hard and soft Lewis acids, respectively, due to their high (low) charge density and small (big) ionic radius.^59^ Therefore, we hypothesize that TBA^+^ and Li^+^ are most likely nonsolvated (low DN number of chloroform) and appear either naked (in the case of TBAClO_4_) or in the form of ion pairs (in the cases of LiClO_4_ and TBAPF_6_) due to the Lewis acid/base strength combinations, and thus, can interact with the polymer chains in the solution. This interpretation agrees well with the EPR results that showed a small but detectable level of free-generated carriers, more substantial for the case of TBAClO_4_ (where TBA is not solvated and not tightly bound to ClO_4_).

After verifying the interactions of cations with polymer chains, we investigated the effect of these interactions on the film’s chemical structure and molecular conformations using Raman and FTIR spectroscopy studies. Raman spectra of the polymers in *as-cast* films are presented in Figure 3c, and their respective peaks attribution is listed in Table S2. The vibrational assignments were based on our previous work on an n-type analog of P75.^60^ The region between 1100 and 1800 cm^–1^ is characteristic of the resonant region of carbon bonds in the conjugated backbone, and the low energy region (<1100 cm^–1^) is attributed to the side chains. We observe three high-intensity peaks associated with the collective C=C–C bonds stretching/shrinking confined on the T2 unit (1457 cm^–1^), the NDI unit (1407 cm^–1^), and the delocalized vibrations of the NDI-T2 monomer (1431 cm^–1^) (marked with a circle in the figure).^50^ These Я-modes describe the direction along which the C=C–C bond vibrations are most effective, and as such, they are the most sensitive to the π-electron perturbations.^51^ The peaks around 1710, 1612, and 1574 cm^–1^ are attributed to symmetric stretching vibrations of C=O, C=C, and C=N of the NDI unit (marked with a circle in the figure), respectively, while the peak at 1541 cm^–1^ is associated with antisymmetric stretching of C=N. Finally, other minor vibrations around 1115, 1231, and 1300 cm^–1^ are related to CH and CH_2_ modes. We generally observe subtle but distinct changes in the Raman spectra upon salt introduction. For films cast from salt-containing solutions, we observe a general shift toward a higher wavenumber of the C=C–C (T2) peak. At the same time, the C=C–C (NDI) is barely affected, suggesting that the salt cation interacts preferably with the T2 unit (Figure 3c, inset-left). These interactions between the T2 unit and the salt additives are expected to lead to a reduced electron localization on the T2 ring, thus increasing the electron delocalization along the conjugated backbone, due in part to the greater planarity of the backbone.

Consequently, the nodes in the π-electron density are reduced, expected to improve the charge mobility. Furthermore, after normalization of the peak intensities to that of the C=N (1574 cm^–1^), we observe a general increase in the intensity of the C=O peak (1710 cm^–1^) for all salt combinations (Figure 3c, inset-right), suggesting a strong interaction of the salt additives with the oxygen of the NDI, associated with its highly polar nature. The widths of Raman peaks can provide structural information, as it is characteristic of the distribution of molecular orientations in the sample.^61^ We notice a slight broadening of the C=O peak in the presence of salts (Figure 3c, inset-right). Thus, the increased width of the C=O in the presence of the salt additives indicates a broader distribution of the different conformations of the polymer chains. TBAClO_4_ leads to a higher intensity of the NDI triplet, as well as a broadening of the C=C (NDI) (1407 cm^–1^), while TBAPF_6_ and LiClO_4_ result in lower NDI triplet intensity and broadening of the C=C (1612 cm^–1^). These findings suggest different interactions of the salts with the P75 backbone, where TBAClO_4_ interactions seem more limited to the NDI unit (C=C and C=O), while the other salts have a broader action (C=C). This perhaps correlates with P75-TBAClO_4_ being the highest-performing material due to the strong localization of charges on the NDI units in NDI-based polymers.^62^ Moreover, given that we could not find evidence of the salt additives being present in *exposed* films, we suggest that these salt/polymer interactions must happen in solution and during film formation. Note that the salt concentration we used (20 mol %) is not high enough to completely disturb the polymer chain conformation as Raman spectra of 5 mol % salt-polymer thin films are identical to the 20 mol % (Figure S14).

We used FTIR spectroscopy to probe further the molecular interactions between the different salts and the polymer backbone (Figure 3d and Table S3). FTIR spectra of the salt-based films show subtle differences compared to the pristine film. In as-cast salt-based films, the out-of-plane C–H bending (T2) at 905 cm^–1^, the in-plane bending vibration of the C–H (NDI) at 1202 cm^–1^, and the antisymmetric C=O (1667 cm^–1^), all shift toward higher wavenumbers (Table S3). These shifts indicate the preferential interactions of the salt additives with the T2 unit and C=O group of the NDI, in line with our Raman observations. The shift toward a higher wavenumber suggests an increased electron delocalization, correlating with our Raman analysis.

**Figure 4:**
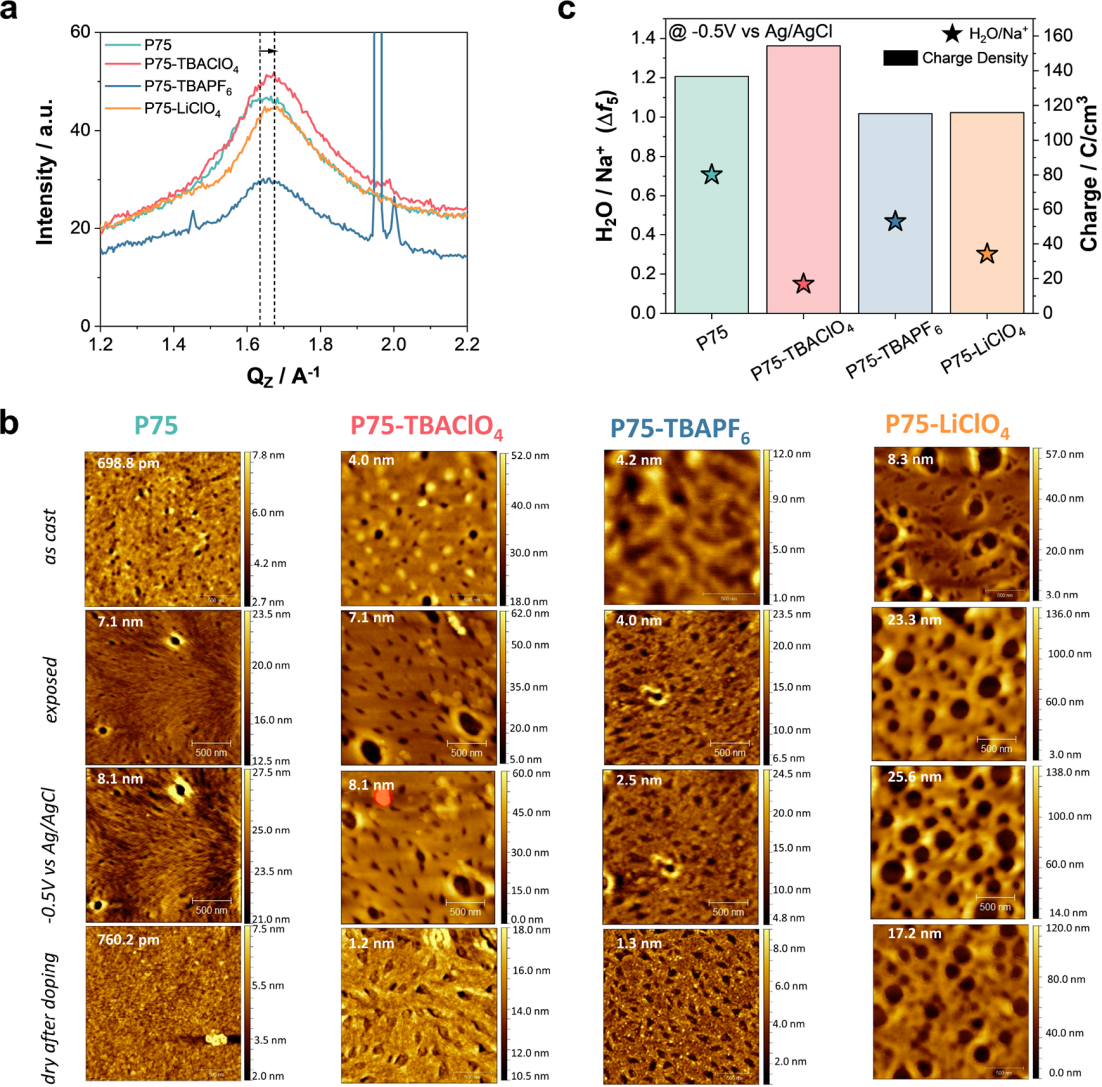
Structure, morphology, and swelling characterization of polymer films. (a) Out-of-plane (*q*_*Z*_) π-peak. The P75-TBAPF_6_ sample is ∼30 nm thick, while the other polymer films’ thickness is ∼50 nm, responsible for the slightly lower intensity for P75-TBAPF_6_. The dotted line serves as a guide to the eye to illustrate the π-lattice contraction occurring in salt-based films compared to the pristine polymer. (b) AFM topography images of the films in various states: dry, swollen in the electrolyte, doped, and dried after doping. Images are 2 μm × 2 μm, and the scale bar is 500 nm. The average surface roughness for each scan is indicated. (c) Calculated density ratio of H_2_O and Na^+^ species injected into the films upon electrochemical doping (−0.5 V vs Ag/AgCl). The corresponding volumetric integrated charge for the first reduction pulse is shown on the right *y*-axis. All EQCM-D measurements were performed using a deoxygenated electrolyte.

Salt additives in the polymer solution significantly improve the device *g*_m_, stemming from higher charge mobility. The salt additives do not seem to generate many more new charges via conventional doping nor reduce the parasitic reactions of the film with O_2_. We can only detect salt cations in *as-cast* films and that they are expelled from the film once immersed in the electrolyte. Although subtle, the changes observed in Raman and FTIR spectroscopies suggest an improved charge delocalization and backbone planarity due to film/salt interactions, which have previously been shown to reduce the π–π distance.^63,64^ We thus sought to understand the impact of the salt additives on film microstructure and analyzed GIWAXS patterns of the polymer films (Figure 4a and Figures S15–S17). First, all polymers are weakly crystalline and adopt a predominantly face-on crystal structure in *as-cast* form (Figure S15). There is no substantial difference in the scattering intensity between these films beyond the difference in thickness, as seen by the line cuts in Figure S16. The presence of salt does not increase or decrease the absolute crystallinity, despite the significant differences in OECT mobilities measured for these films. However, the neat polymer film has a peak at 1.64 Å^–1^, while the salt cast films have a higher lattice position, around 1.67 Å ^–1^ (Figure 4a). This shift suggests a small but distinct contraction (≈ 2.3%) of the π–π spacing in all of the samples cocast with salt compared to the neat polymer film. This contraction correlates with the improved charge transport in salt additives-based OECTs, as the π–π distance has previously been shown to be indicative of transport performance,^65^ and confirms our hypothesis based on Raman/FTIR results. Moreover, we observe the presence of an isotropic ring in the 2D GIWAXS plots at 0.61 Å^–1^, particularly obvious for the P75-TBAClO_4_ sample, which we assign to the salt (or cations) crystals themselves (Figure S17). This feature is consistent with nanocrystals of TBA salts with a random distribution within the film.^66^ We suggest that as the polymer is cast, the salt crystallizes within the polymer bulk during film formation.

However, we do not observe such peaks for the other salt-cast polymers. Nonetheless, given our previous XPS and SIMS results, we can generalize our observation to TBAPF_6_ and LiClO_4_, precipitating to some extent within the polymer during film formation. Furthermore, the GIWAXS profile of P75-TBAClO_4_ for *exposed* films confirms that these salt crystals are no longer present after electrolyte exposure as the salt peak at 0.61 A^–1^ in P75-TBAClO_4_ disappears, consistent with the XPS/SIMS measurements (Figure S17). All the changes observed are quite subtle despite significant consequences on the transistor performance and conductance, similar to the results seen for poly(3-hexyl thiophene) doped with lithium bis(trifluoromethane sulfonyl)imide (LiTFSI).^67^

We, however, see very distinct surface morphologies for salt-cast films. The salt-based as-cast films display an increased surface roughness, porosity (Figure 4b), and broader height distribution compared to the pristine film (Figure S18). The pristine film has the most uniform and compact surface, while P75-LiClO_4_ presents the most porous and roughest surface of the set. The TBA-based films show similar surface roughness. When *exposed* to the electrolyte, the pristine film exhibits drastic changes in its roughness, increasing by 1 order of magnitude, with the surface gaining widened features (Figure S18). The TBA-cast films undergo a relatively moderate morphological change and demonstrate similar surfaces upon exposure to the electrolyte, showing increased porosity. We can associate these changes in morphology (porosity and height distribution) upon electrolyte exposure with the salts leaving the polymer films. Applying a doping potential (−0.5 V vs Ag/AgCl) does not seem to induce further morphological changes for the salt-bearing films. At the same time, the pristine polymer displays significant changes in its relative height distribution (Figure S18). Once these films are dried after doping, we observe more pores for the salts-based films compared to their *as-cast* conditions (Figure 4b). P75-LiClO_4_ displays a rougher surface compared to its *as-cast* film, while the TBA-based films exhibit smoother surfaces, and P-75 shows not much change. We note the similarities of the changes and final morphology for the TBA-based films, yet they present different OECT performances. This result highlights the differences in interactions of the TBA cation with the polymer as a function of its coanion, as we discussed with the HSAB theory and Raman spectra. Furthermore, the increased porosity of films has previously been linked with faster device operation,^68,69^ in line with our response-time measurements.

Lastly, we sought to understand whether the film cycling and shelf life stability can be linked to its morphology and ion uptake and release profile. The rougher and more porous surface highlighted in the AFM images of salt-bearing films may lead to enhanced ion uptake and transport. Such a porous morphology may allow ion uptake without significant distortion of film morphology.^68,70^ One method to elucidate the films’ ion uptake/release profile upon electrochemical doping is electrochemical quartz crystal microbalance with dissipation monitoring. The e-QCMD measures the change in frequency of a film coated on a quartz-coated crystal under bias and thus provides a means to relate the combined weight of water and ions injected into the polymer films to the charge generated. Consequently, it can give an estimate of the polymer ion-to-electron coupling efficiency.^22^ First, we measured the passive swelling (i.e., in the absence of electrical bias) of the different thin films upon exposure to the electrolyte (Figure S19). While all films swell to some extent (5% to 37%), it is not easy to interpret these results as we have shown the salt additives exiting the films upon electrolyte exposure. We then evaluated the active swelling, i.e., under electrochemical biasing, upon three consecutive electrochemical doping/dedoping cycles (Figure S20). We expect a similar amount of electrochemical doping for all the films, given that they have similar open circuit (*V*_OC_) values (Table S4) and similar C* values. Overall, all the polymers show a low level of active swelling (<10%) when compared to their passively swollen thickness (Table S4), in line with the relatively unchanged morphologies observed under electrochemical doping for salt-bearing films. Figure S20 illustrates the mass changes for each reduction cycle, showing that P75-TBAClO_4_ uptakes the least amount of mass out of the series. Note that this film is also our top OECT performer.

Figure 4c represents the calculated charge for the first reduction pulse, where P75-TBAClO_4_ (168.2 C/cm^3^) produces the highest amount of charge, followed by the pristine polymer P75 (151.8 C/cm^3^), P75-TBAPF_6_ (135.3 C/cm^3^), and finally P75-LiClO_4_ (127.1 C/cm^3^). We then calculate the Na^+^ density, assuming that one cation couples with one electron in the film.^71^ The cation mass we estimate from the integrated currents is lower than the mass we measured using e-QCMD. The extra mass is attributed to water dragged into the film alongside the cation. The ratio of water to cation (H_2_O/Na^+^) is thus a representation of how much water enters the film during doping, represented in Figure 4c. We find a lower H_2_O/Na^+^ ratio for salt-bearing films. Excess water uptake has been associated with the lower mechanical stability of such films under biasing.^72^ A lower amount of water for the salt-based films may be the reason for the long-term stability of their devices. However, we emphasize that this hypothesis is based on several assumptions that may not apply to every polymer similarly.

The improved mixed transport and cycling stability in the salt-based films could be tied to the porosity observed in AFM. However, the H_2_O/Na^+^ ratio seems independent of the pore size (P75-TBAClO_4_ and P75-TBAPF_6_ show similar pore sizes, yet P75-TBAClO_4_ displays a lower ratio). We thus analyzed the connectivity χ of these pores using the AFM images through the Minkowski function distribution (Figure S21). The Minkowski connectivity graphs describe the connectivity of spatial patterns in relation to changes in morphology and surface geometries.^73^ We find that P75-TBAClO_4_ and P75-LiClO_4_ present a more interconnected pore network than P75-TBAPF_6_ and the pristine film, which might be the reason for the lower amount of water these films take up. Furthermore, the apparent lower interconnectivity of the pristine P75 surface reminds us of the more significant morphological changes that this film undergoes upon electrochemical doping and can be responsible for its low cycling stability.

## Conclusion

This work shows that the steady-state performance, switching on and off speed, shelf life, and operational stability of n-type OECTs can be simultaneously improved by casting the n-type films from salt-blended solutions. Despite the extensive characterization discussed above, we could not identify a single significant factor that we can point to as the source of the observed electron mobility improvement. This leaves us to conclude that the improved performance is caused by several subtle differences in electronic structure combined with an aspect of the morphology which is notoriously difficult to characterize, such as amorphous regions, tie chains, or crystal interconnectivity. The importance of our approach lies in its simplicity. We add the salts in ambient conditions to the polymer solution. The salt ions mainly interact with the backbone so that electronic charge carrier mobility and ion uptake are improved. XPS and SIMS demonstrate the presence of the salt cation within the film in *as-cast* conditions only and that they diffuse out of the films upon electrolyte exposure. EPR spectroscopy suggests that the salt additives only marginally dope the polymer, in agreement with the similar capacitances measured for salt-bearing and pristine films. Raman and FTIR spectroscopies illustrate the preferential interactions of the salt additives with the T2 unit and C=O group of the polymer, indicating an increased charge delocalization and better planarity of the backbone. GIWAXS measurements show a small but distinct contraction of the π-lattice. We observe a different, porous morphology for the salt-based films, with widened pores as the salts exit the films. In situ AFM measurements display significant changes in the pristine film morphology upon electrochemical doping, while the salt-bearing film morphology is barely affected. These results hint at greater mechanical integrity of the salt-based films, a possible origin for their improved stability. Moreover, these films take up less water during electrochemical doping, which might lead to a prolonged shelf life and cycling performance. We hypothesize that the different concentrations and solubility between the salt additives and polymer chains in chloroform cause the precipitation of salt nanocrystals in the film during the fast solvent evaporation, which is then responsible for morphological and molecular rearrangements. The introduction of salt additives is an easy-to-use technique alternative to n-dopants to enhance the performance and stability of n-type materials. The effect of salts in the casting solution may be enhanced by using solvents offering different salt and polymer solubilities as well as through annealing of the films. We believe that the salt additives strategy introduced in our work would inspire other approaches that focus on optimizing film morphology for enhanced mixed charge transport, elevating the performance of n-type materials.

## Supporting Information Available

OECT output characteristics, materials energy levels and UV–vis-NIR absorption spectra, volumetric capacitance determination, transistor response time and operational stability, EPR spectra, O_2_ sensitivity of n-type films, XPS and SIMS signals of n-type films, AFM and GIWAXS data, and QCM-D traces, models, and analysis of n-type films (PDF)
