# Supplementary material for: Salts as Additives: A Route to Improve Performance and Stability of n-Type Organic Electrochemical Transistors
Source: ACS Mater Au. 2023 Mar 6;3(3):242–54. doi: 10.1021/acsmaterialsau.2c00072 (PMC10176614; doi:10.1021/acsmaterialsau.2c00072)
Supplement: Supplementary file 1 — mg2c00072_si_001.pdf [file mg2c00072_si_001.pdf]

SUPPORTING INFORMATION

Salts as additives: A route to improve performance and stability of n-type organic electrochemical transistors

David Ohayon[1], Lucas Q. Flagg,[2] Andrea Giugni,[3] Shofarul Wustoni[1], Ruipeng Li,[4] Tania C. Hidalgo Castillo,[1] Abdul-Hamid Emwas,[5] Rajendar Sheelamanthula,[6] Iain McCulloch,[6, 7] Lee J. Richter,[2] Sahika Inal[1*]

[1] Organic Bioelectronics Laboratory, Biological and Environmental Science and Engineering Division, King Abdullah University of Science and Technology (KAUST), Thuwal 23955-6900, Saudi Arabia.

[2] Materials Science and Engineering Division, National Institute of Standards and Technology (NIST), Gaithersburg, Maryland 20899, United States.

[3] Department of Physics, Università degli Studi di Milano, Via Celoria 16, I-20133 Milano, Italy.

[4] National Synchrotron Light Source II, Brookhaven National Laboratory, Upton, New York 11973, United States.

[5] Core Labs, KAUST, Thuwal, 23955-6900, Saudi Arabia.

[6] Physical Sciences and Engineering Division, KAUST, Thuwal 23955-6900, Saudi Arabia

[7] Department of Chemistry, Chemistry Research Laboratory, University of Oxford, Oxford OX1 3TA, U.K.

*Corresponding author: sahika.inal@kaust.edu.sa

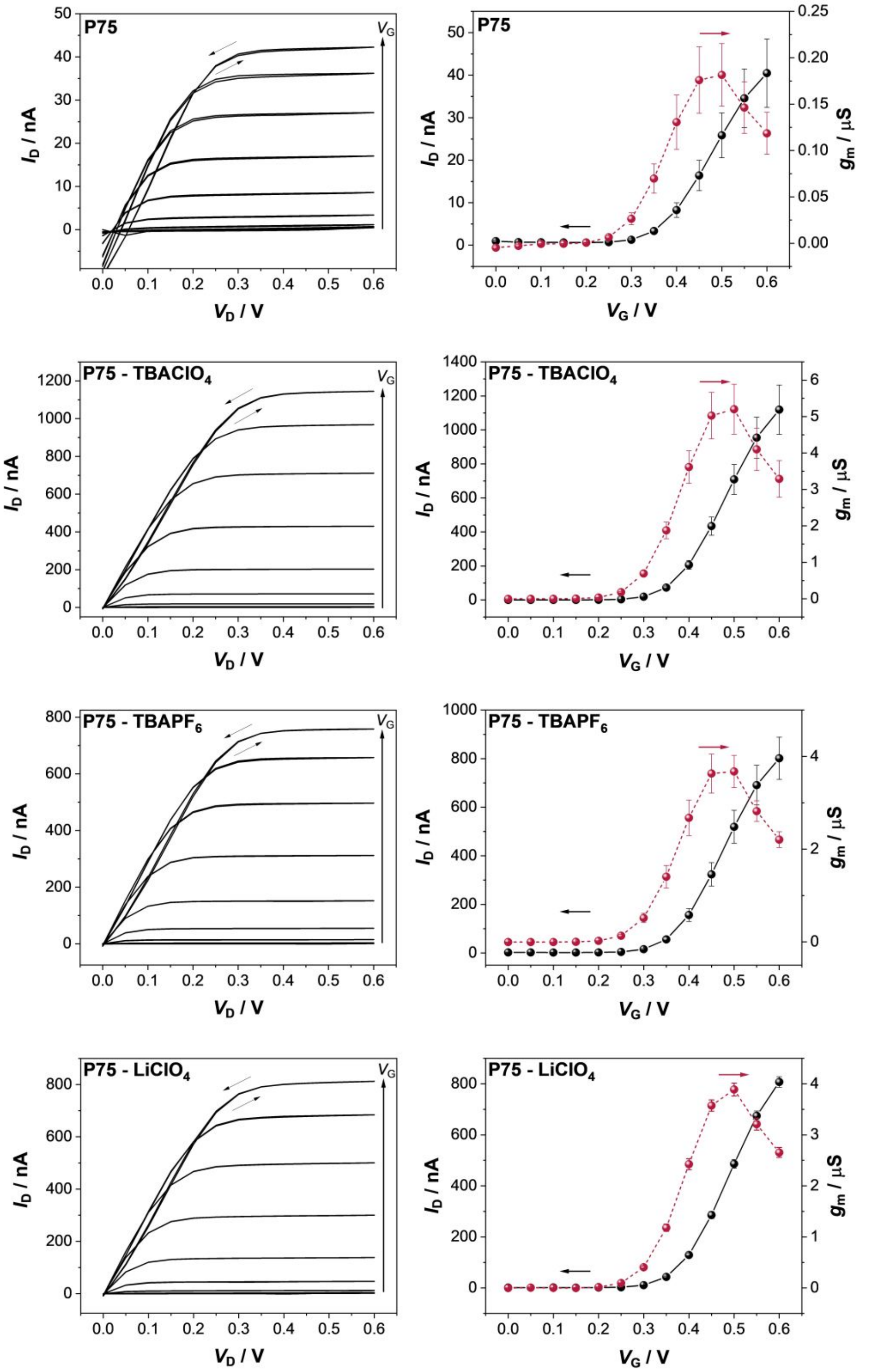

Figure S1. Output and transfer curves, and the transconductance vs. gate voltage plots. Arrows indicate the scan direction. Transfer curves were obtained at V_D = 0.6 V. The scan rate was 100 mV/s. Error bars represent the standard deviation measured from at least six different devices. Average channel thicknesses were 60 nm, 130 nm, 175 nm, and 180 nm for P75, P75-$TBAClO_4$, P75-$TBAPF_6$, and P75-$LiClO_4$, respectively.

Table S1. Energy levels of P75, P75-$TBAClO_4$, P75-$TBAPF_6$, and P75-$LiClO_4$ measured using photoelectron spectroscopy.

Polymer	IP (eV)	EA (eV)	Band-gap (eV)
**P75**	5.59	4.22	1.37
**P75-$TBAClO_4$**	5.59	4.27	1.32
**P75-$TBAPF_6$**	5.59	4.23	1.36
**P75-$LiClO_4$**	5.58	4.23	1.35

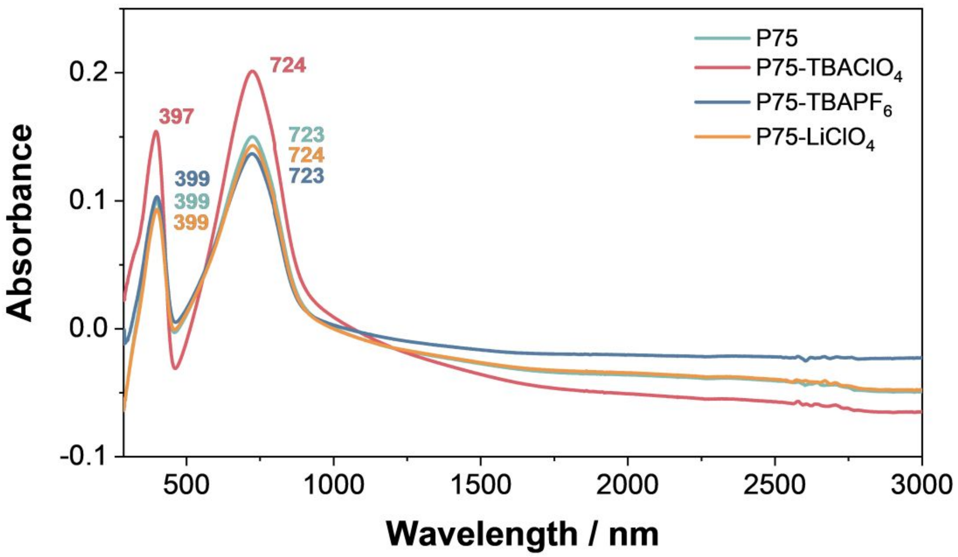

Figure S2. UV-VIS-NIR absorption spectroscopy of as cast thin films on ITO. Thin films have an average thickness of 50 nm.

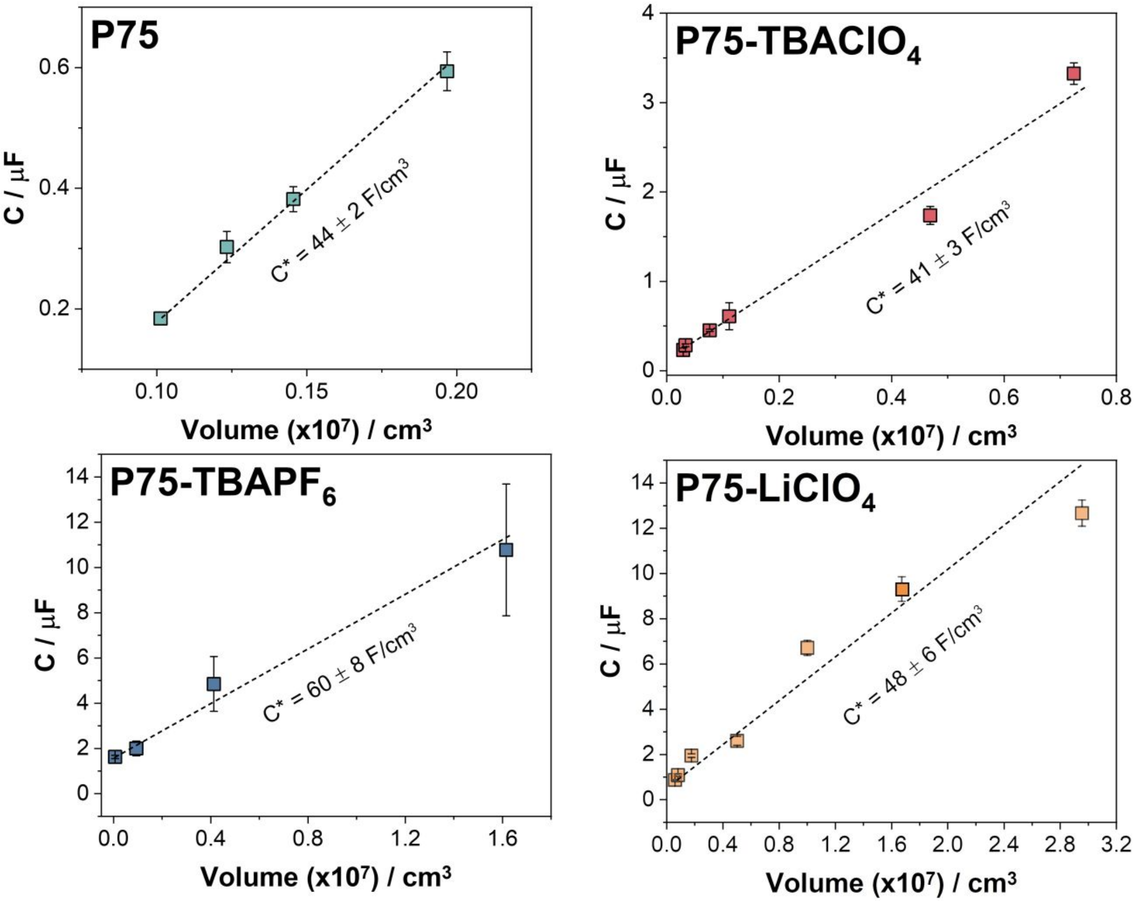

Figure S3. The dependence of film capacitance on its geometry. The capacitance was determined from the electrochemical impedance spectra collected at 0.1 Hz at a DC voltage of -0.5 V vs. Ag/AgCl. Error bars represent the standard deviation over at least 3 different devices per geometry.

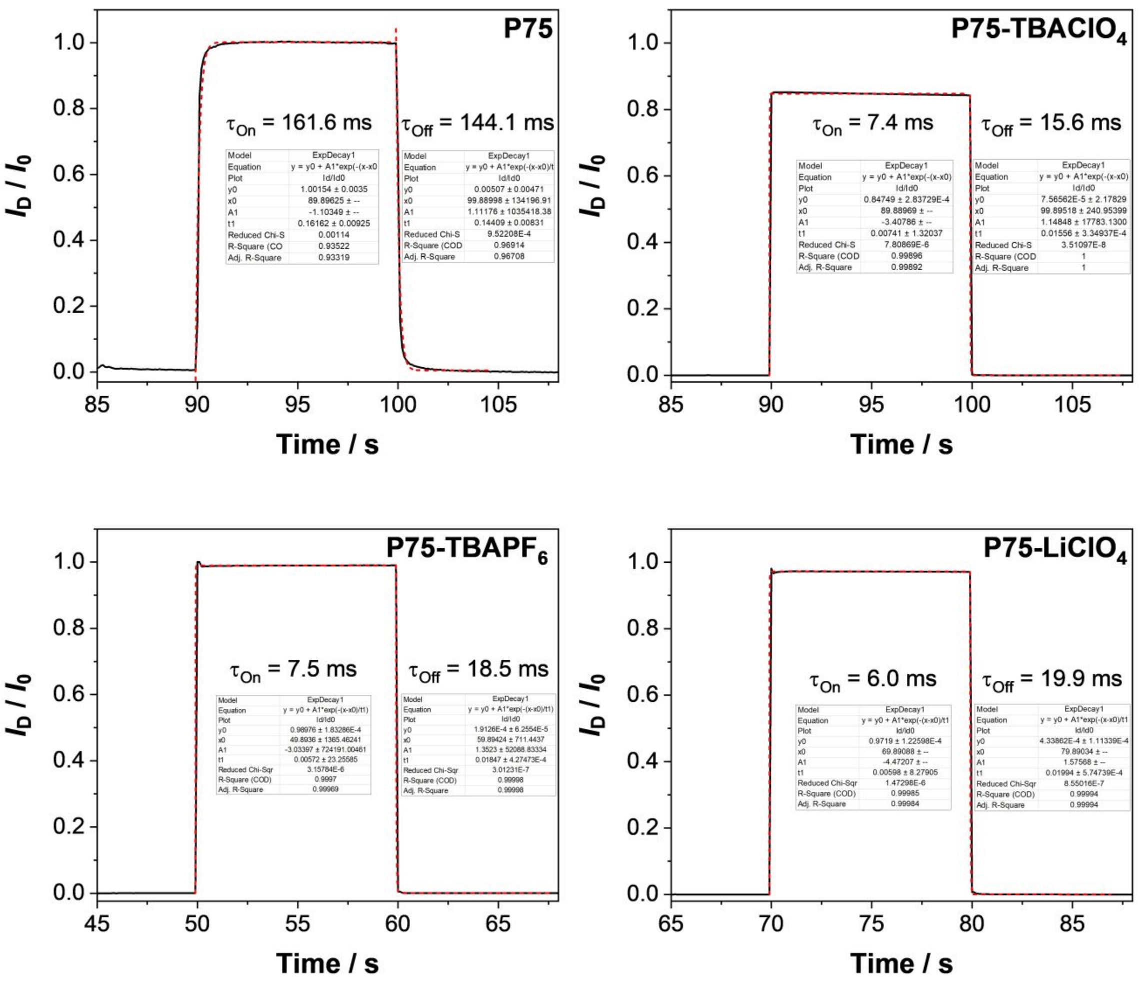

Figure S4. Transient profiles of P75, P75-$TBAClO_4$, P75-$TBAPF_6$, and P75-$LiClO_4$. The drain voltage was at +0.4 V, and a gate voltage pulse of +0.4 V amplitude was applied for 10 s.

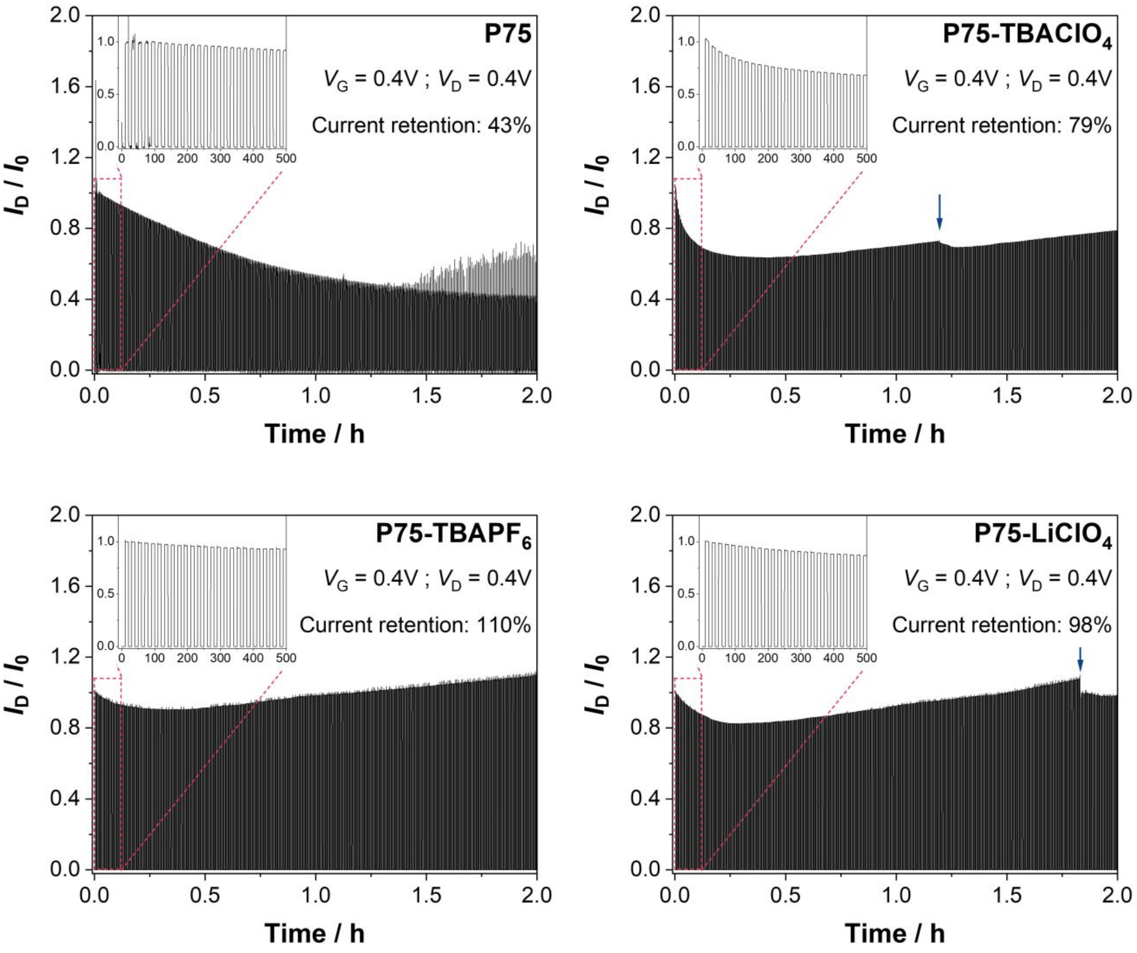

Figure S5. Operational stability of P75, P75-TBAClO$_4$, P75-TBAPF$_6$, and P75-LiClO$_4$. The channel current stability against pulsed gate voltages (V_G = 0.4V) applied for 2 hours. V_G varied between 0 and 0.4 V, with 10-second intervals. V_D was fixed at 0.4V. Note that the arrow indicates the addition of fresh 0.1M NaCl electrolyte. Inset presents the variation of the current in the first 500 seconds of pulsing. I_0 is the drain current corresponding to the first pulse.

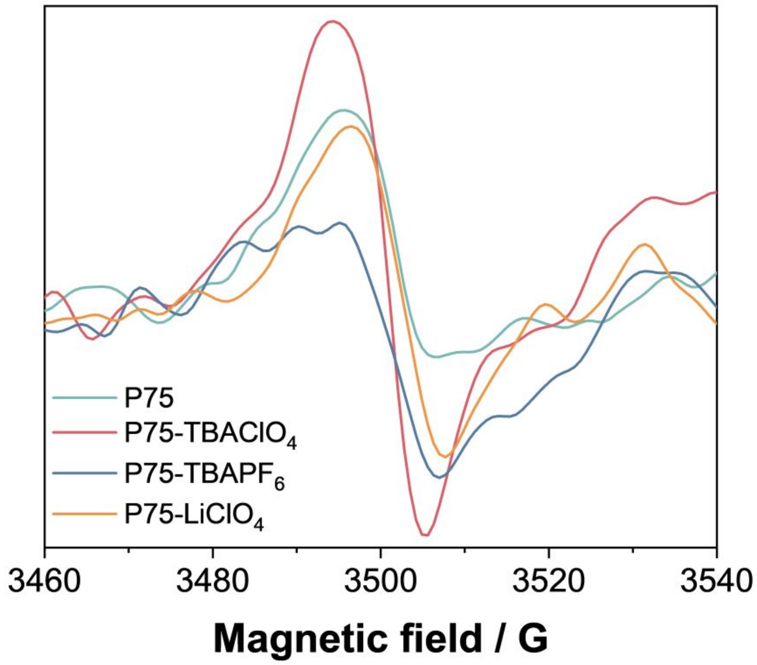

Figure S6. Electron paramagnetic resonance (EPR) spectroscopy for P75, P75-$TBAClO_4$, P75-$TBAPF_6$, and P75-$LiClO_4$ *as cast* films.

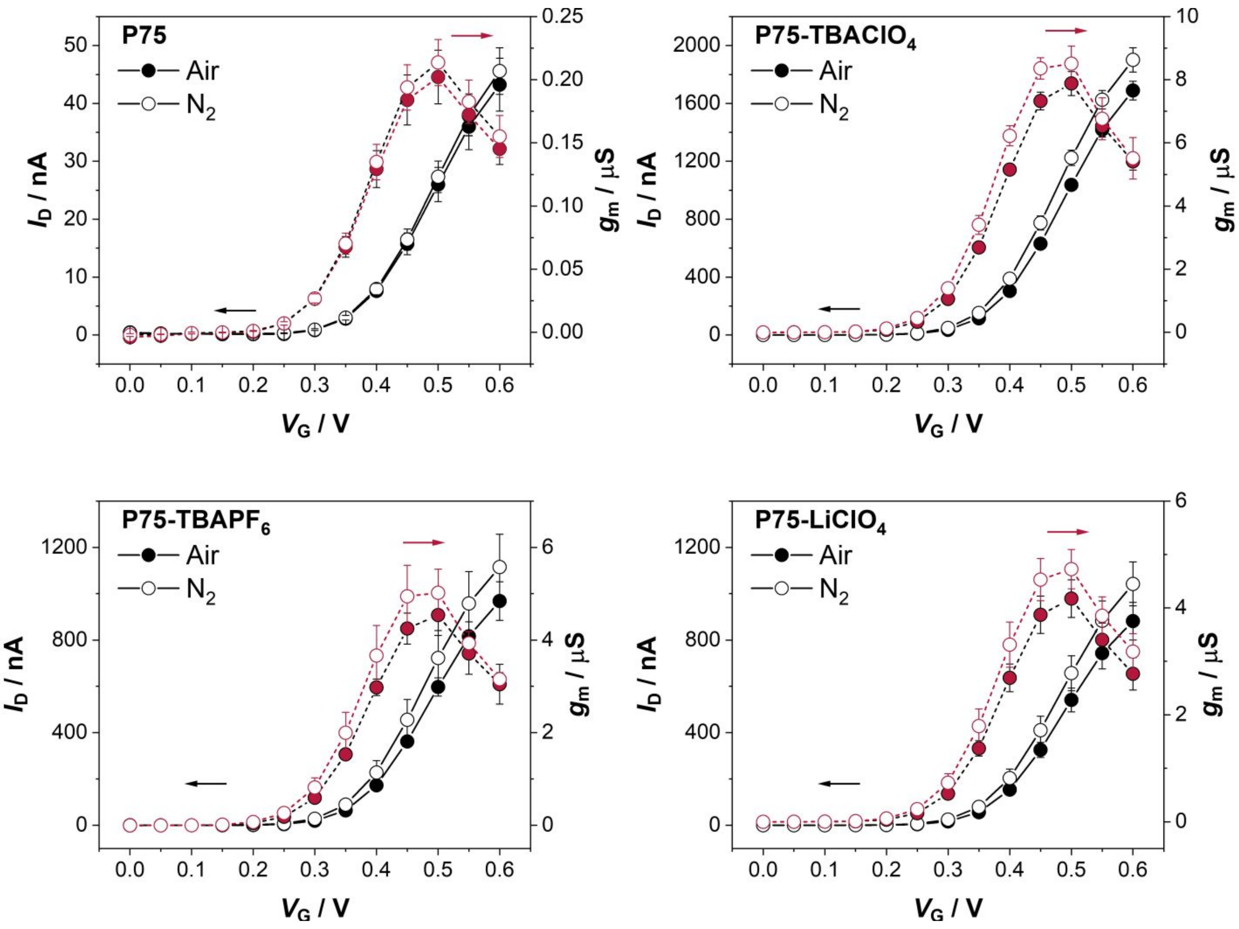

Figure S7. Influence of oxygen on OECT performance for P75, P75-TBAClO$_4$, P75-TBAPF$_6$, and P75-LiClO$_4$. Error bars represent the standard deviation over at least six different devices.

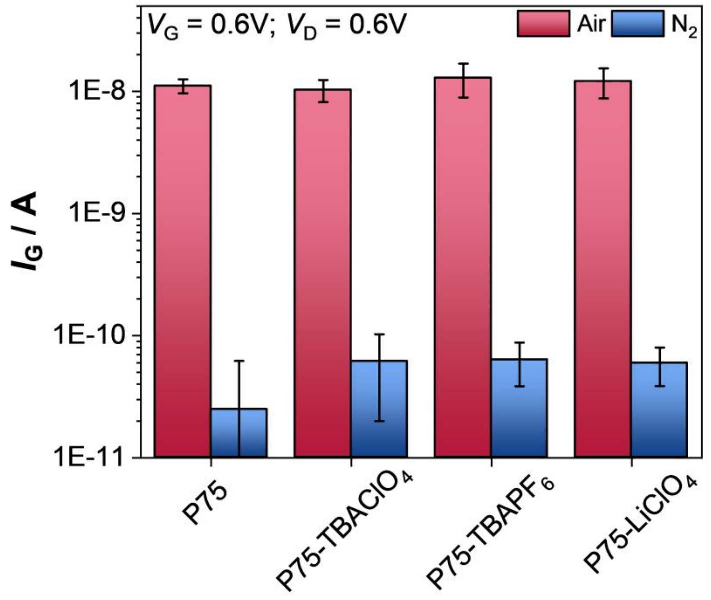

Figure S8. Influence of oxygen on OECT gate current (I_G). The currents were measured at V_G = 0.6 V and V_D = 0.6 V. Error bars represent the standard deviation over at least six different devices.

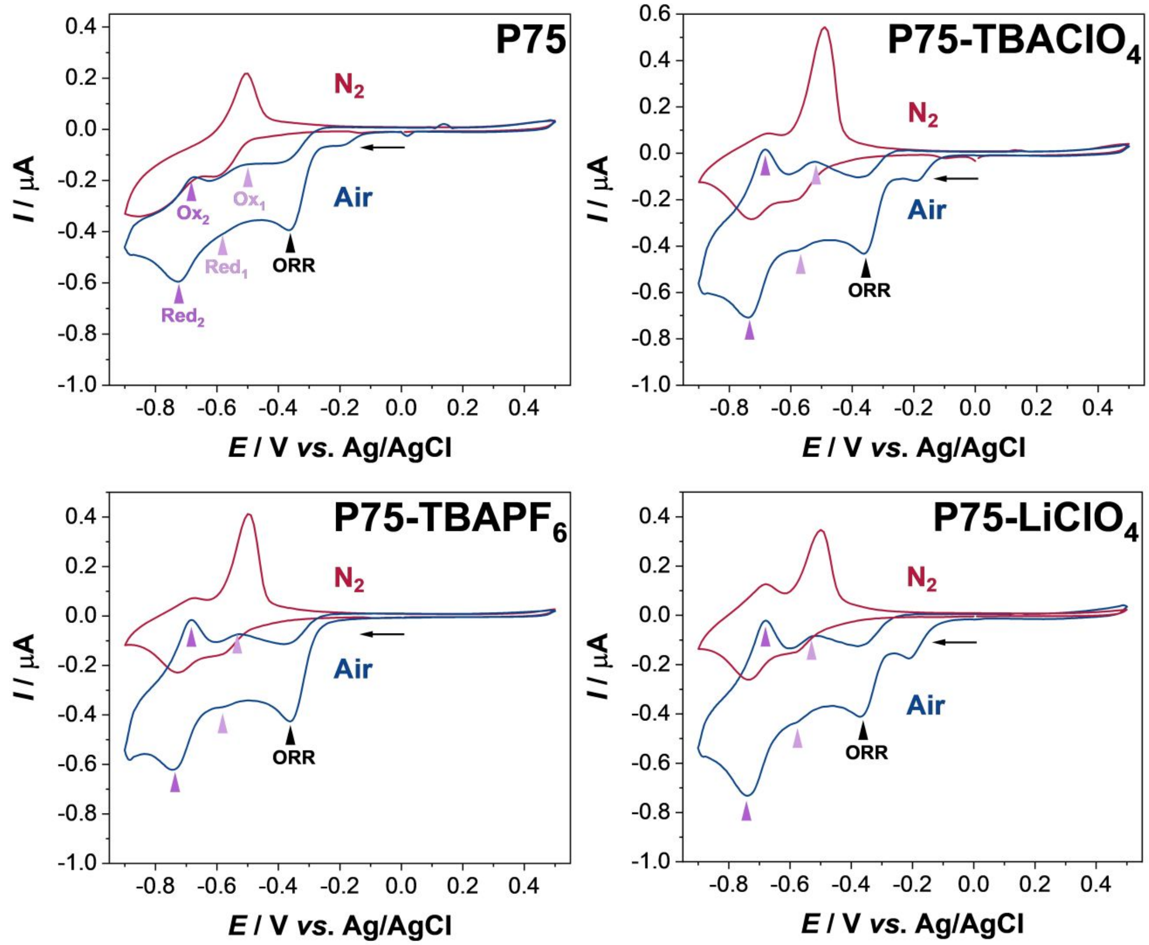

Figure S9. Influence of oxygen on cyclic voltammograms. Arrow indicates the scan direction. Scan rate is 50 mV/s. The average film thickness is 50 nm.

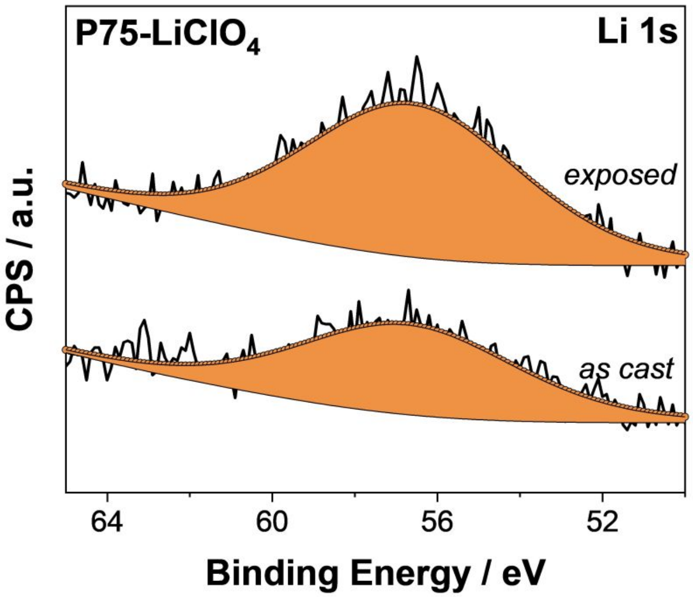

Figure S10. High-resolution Li 1s XPS spectra of P75-$LiClO_4$ *as cast* and *exposed* films. The peak observed here overlaps with (or corresponds to) the Au $5p_{3/2}$ peak which has a typical binding energy of 57.2 eV.

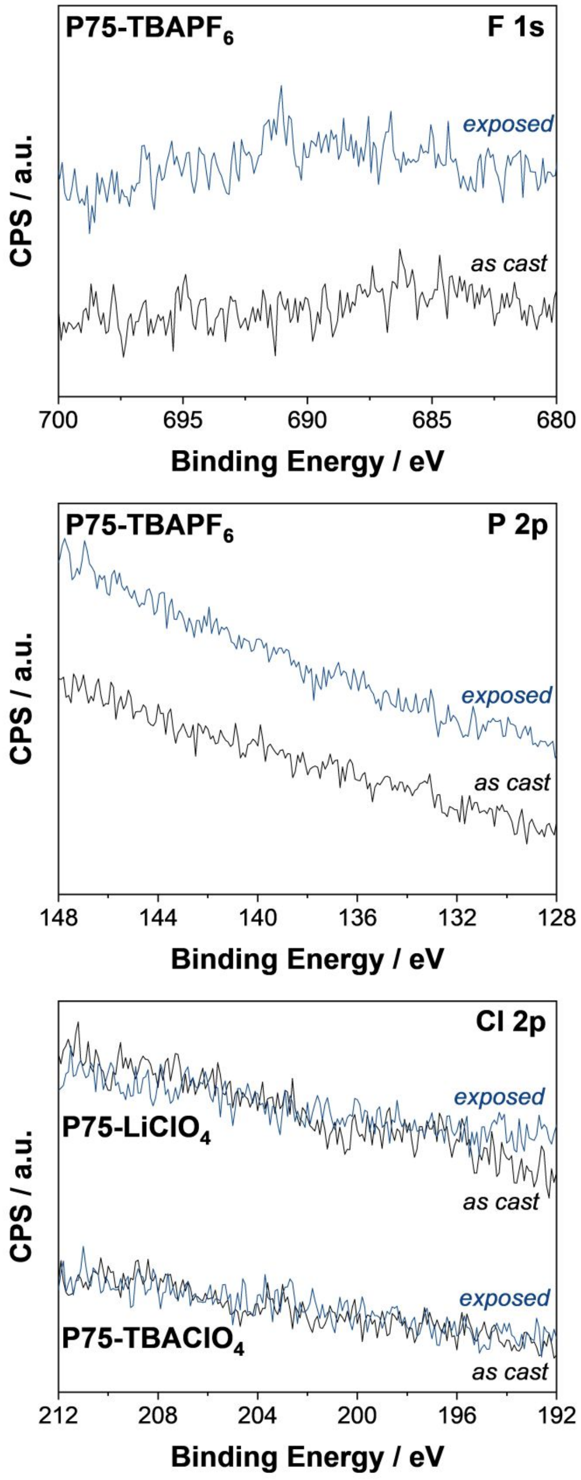

Figure S11. High-resolution spectra of F 1s, P 2p, and Cl 2p spectra of P75, P75-$TBAClO_4$, P75-$TBAPF_6$, and P75-$LiClO_4$ *as cast* and *exposed* films.

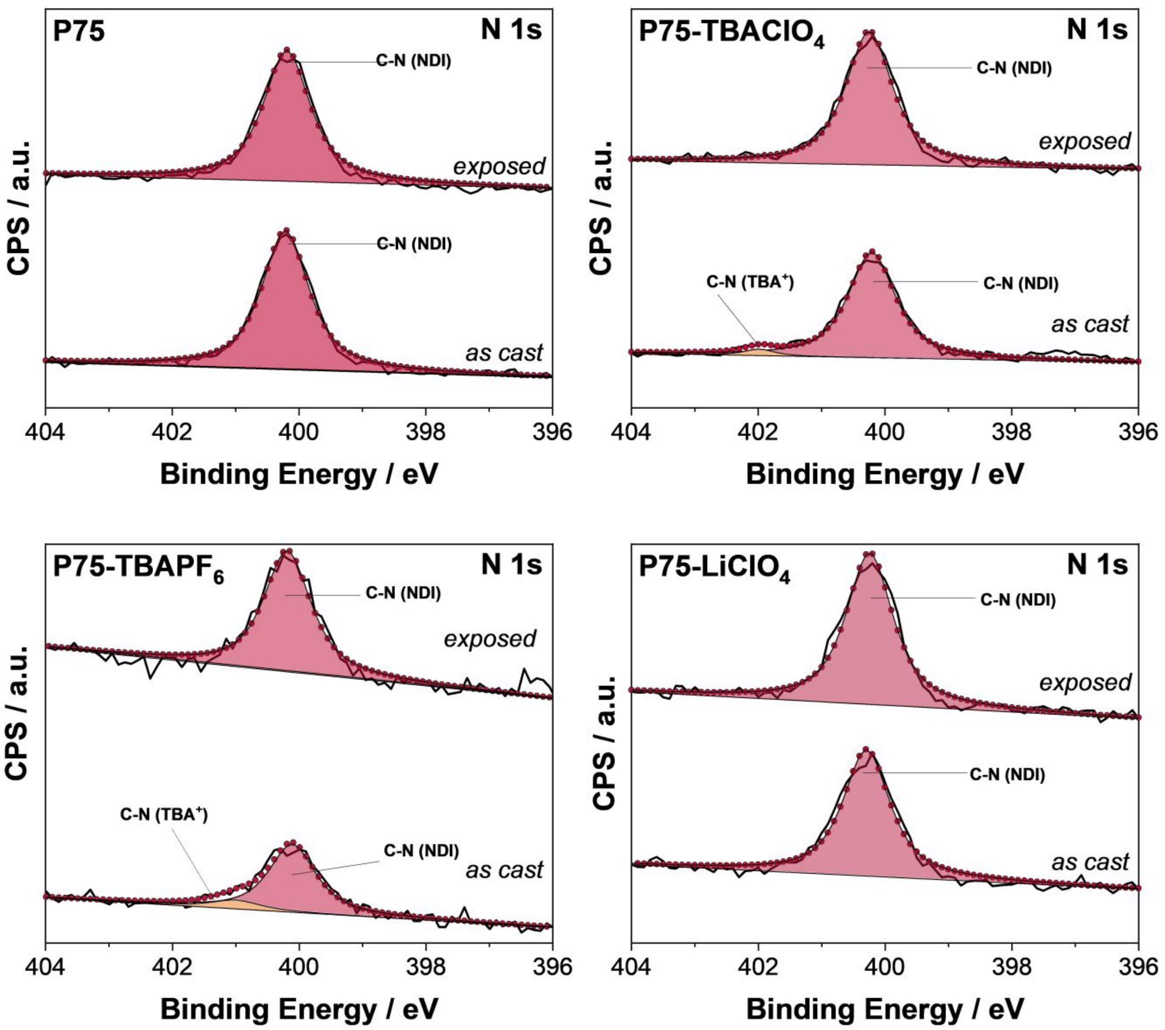

Figure S12. High-resolution N 1s spectra of P75, P75-TBAClO_4, P75-TBAPF_6, and P75-LiClO_4 *as cast* and *exposed* films.

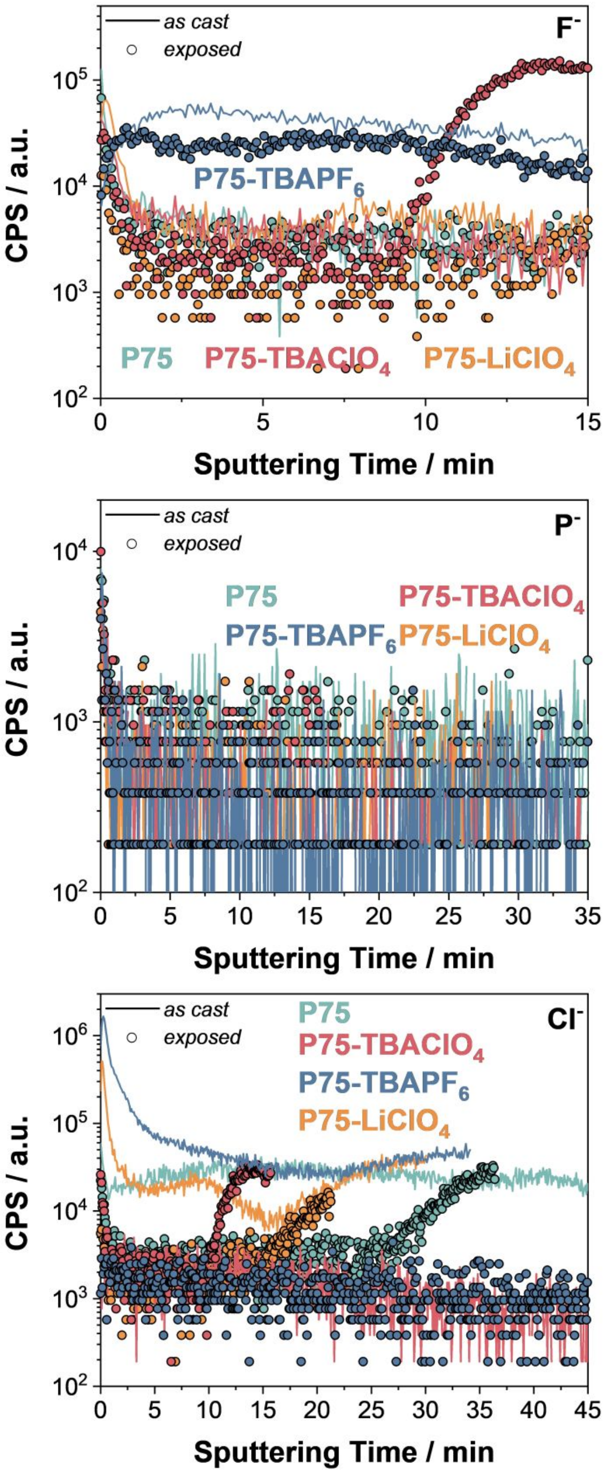

Figure S13. Secondary ion mass spectrometry data showing F$^-$, P$^-$, and Cl$^-$ signals in *as-cast* and *exposed* thin films.

Table S2. Raman peaks attribution for P75, P75-TBAClO$_4$, P75-TBAPF$_6$, and P75-LiClO$_4$ *as cast* films.

	Wavenumber (cm^{-1})									
as cast films	CH and CH$_2$ modes			C=C-C (NDI)	C=C-C (NDI-T2)	C=C-C (T2)	C=N asym	C=N	C=C	C=O
P75	1115.47	1231.12	1300.8	1407.7	1428.49	1458.22	1541.65	1574.16	1612.09	1710.83
P75-TBAClO$_4$	1113.59	1228.33	1307.41	1407.4	1432.1	1459.12	1548.46	1574.45	1613.56	1709.38
P75-TBAPF$_6$	1113.9	1231.73	1302.82	1405.89	1433.01	1459.72	1544.02	1574.93	1611.51	1713.14
P75-LiClO$_4$	1113.27	1230.81	1308.94	1407.09	1430	1459.12	1544.61	1573.27	1613.56	1712.56

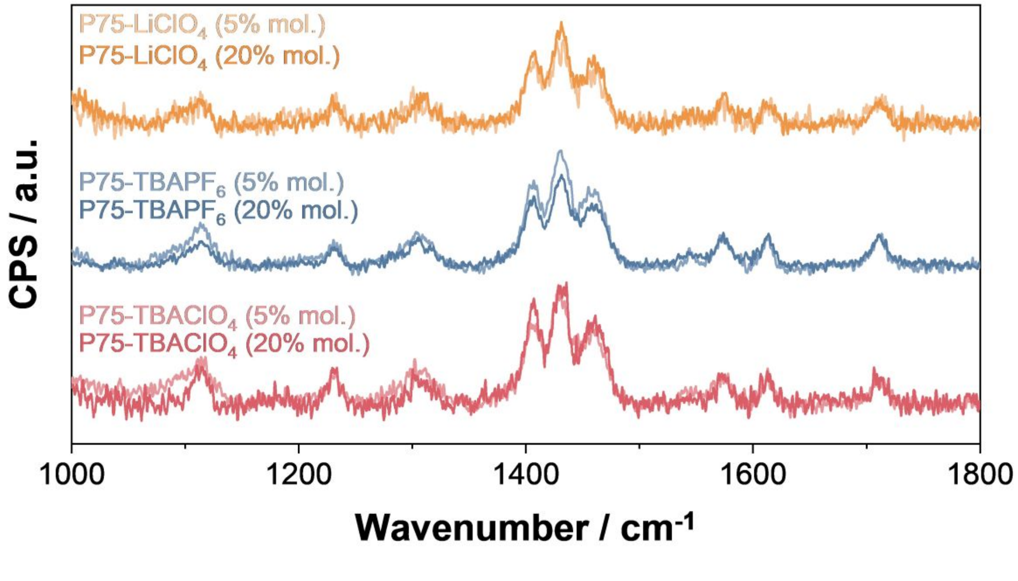

Figure S14. Raman spectra of P75-TBAClO$_4$, P75-TBAPF$_6$, and P75-LiClO$_4$ *as cast* films with 5% mol and 20% mol salt concentration.

Table S3. FTIR peaks attribution for P75, P75-$TBAClO_4$, P75-$TBAPF_6$, and P75-$LiClO_4$ *as cast* films.

	Wavenumber (cm^{-1})			
	P75	P75-$TBAClO_4$	P75-$TBAPF_6$	P75-$LiClO_4$
CH_2 anti-symmetric	2921.8	2918.9	2921.8	2921.8
CH_2 symmetric	2855.3	2859.1	2855.2	2855.2
C=O symmetric	1709.7	-	-	1716.4
C=O anti-symmetric	1667.2	1668.2	1668.2	1668.2
C=C/C-C stretching	1569.8	1568.9	1569.8	1569.8
CH_2 bending	1439.7	1435.8	1436.8	1436.8
C-H bending	1318.2	1314.3	1317.2	1319.1
CH_2 wagging	1266.1	1264.2	1265.1	1266.1
C-H (q_r) bending	1201.5	1208.2	1206.3	1203.4
C-H (q_z) – T2	905.5	908.8	914.1	907.4
C-H (q_z) bending	792.6	790.7	792.6	795.5
C-S (q_r) bending	722.2	-	683.7	718.9

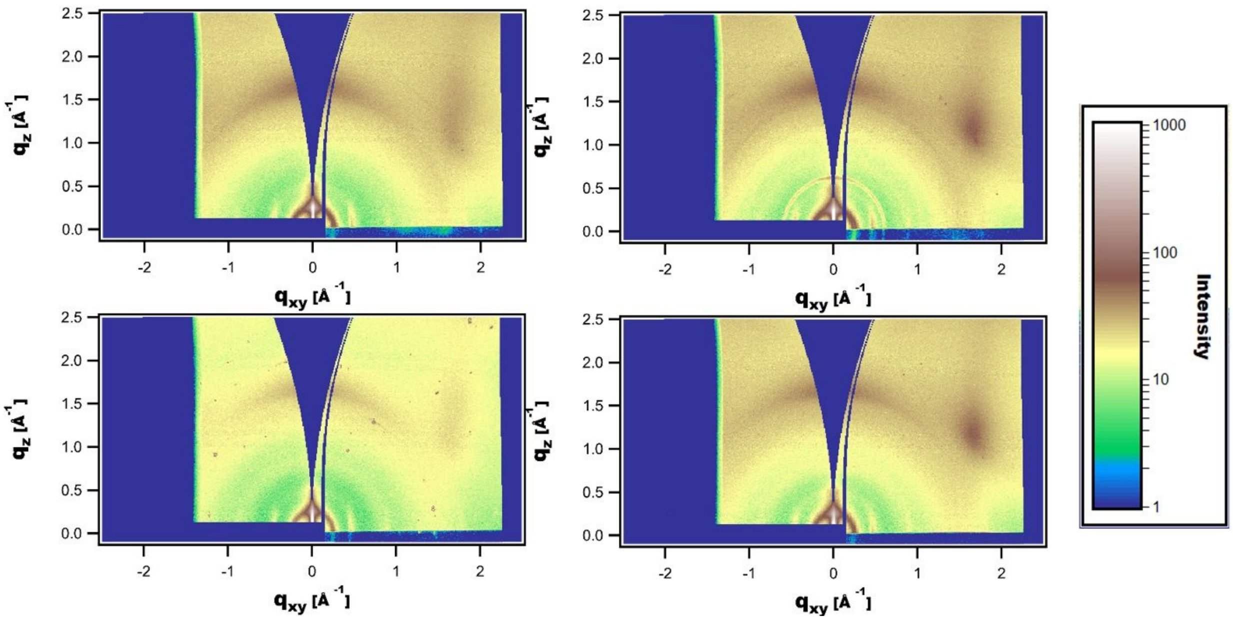

Figure S15. 2D GIWAXS plots of as-cast P75, P75-$TBAClO_4$, P75-$TBAPF_6$, and P75-$LiClO_4$ films.

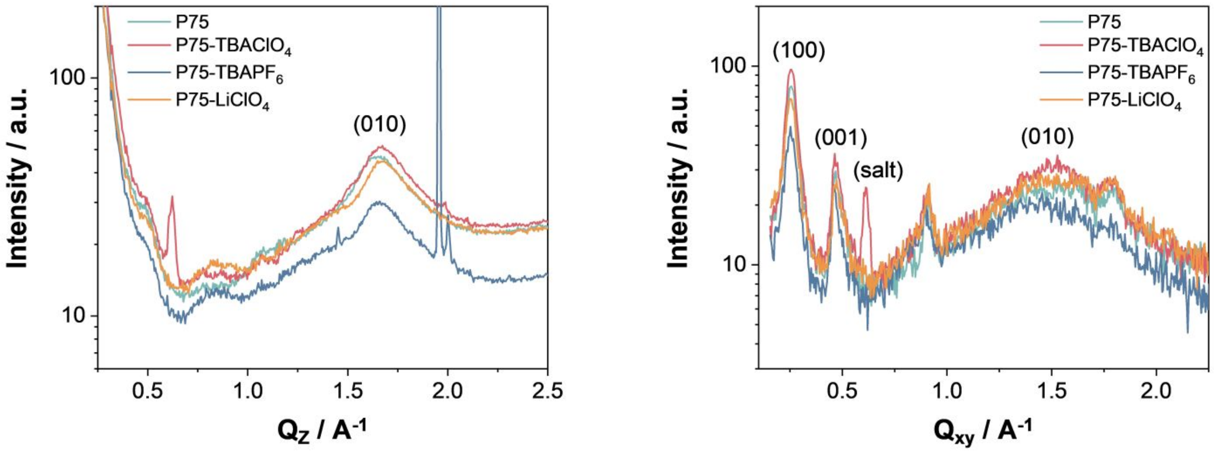

Figure S16. Out-of-plane (q_Z) and in-plane (q_r) line cuts for P75, P75-$TBAClO_4$, P75-$TBAPF_6$, and P75-$LiClO_4$ *as cast* films.

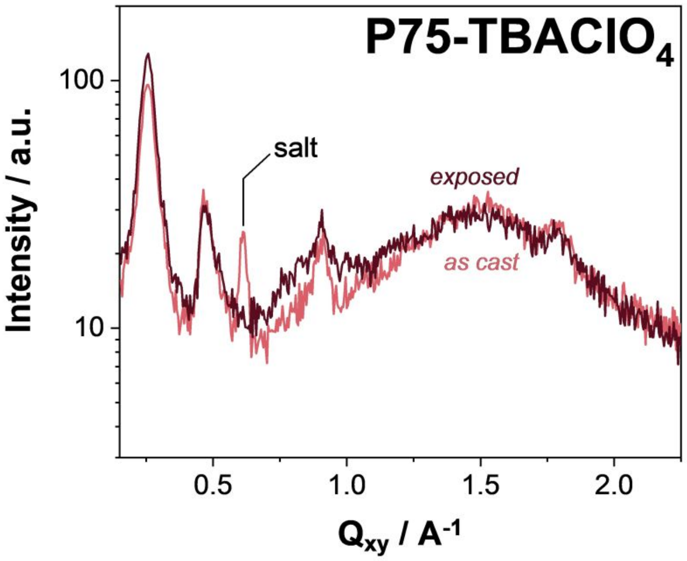

Figure S17. In-plane (q_r) line cut of P75-TBAClO$_4$ *as cast* and *exposed* thin films. The disappearance of the salt peak at 0.61 A^{-1} shows that the salt species leave the film in the testing conditions.

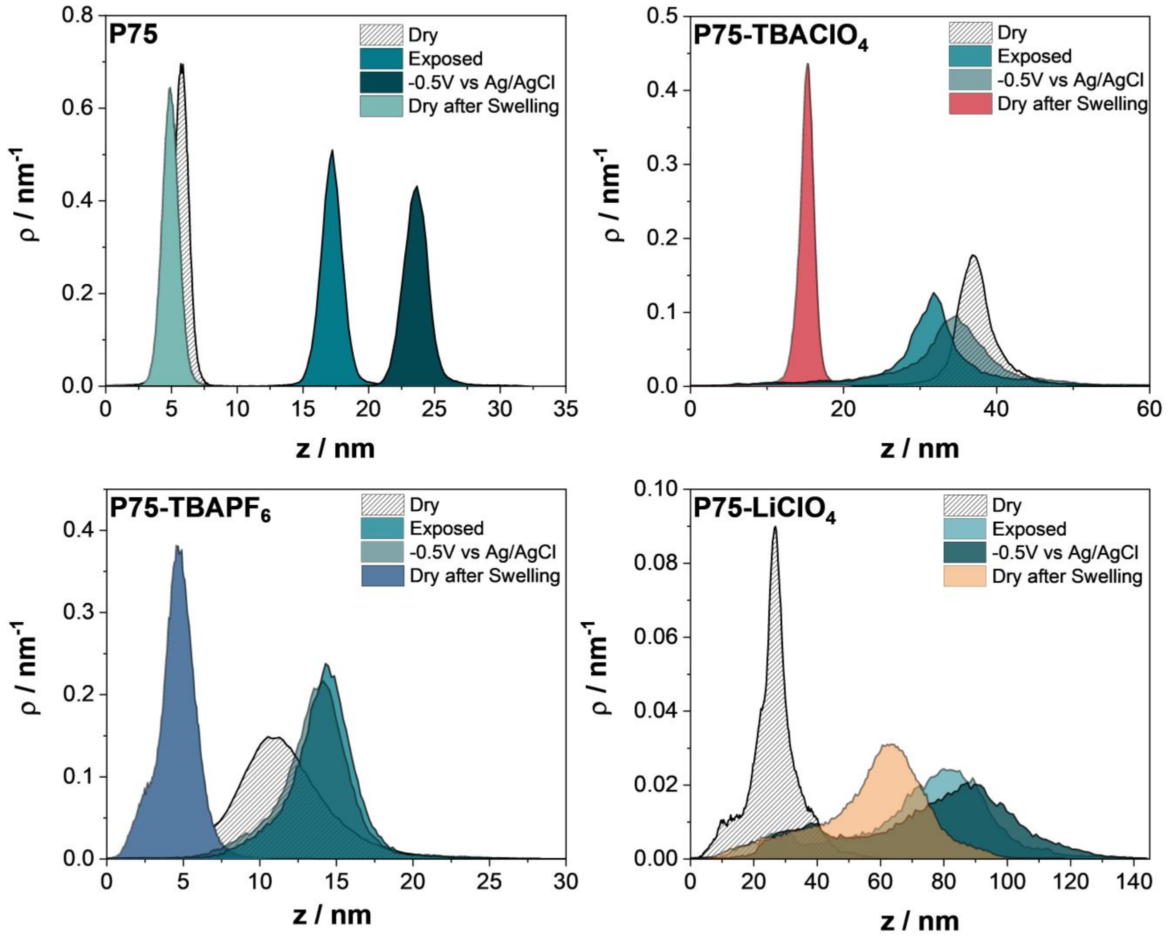

Figure S18. The height distribution curve for each AFM image. The height distribution size (X-axis) represents the relative size feature of each individual scan due to the lack of a common zero baseline. The pristine polymer displays a reversible surface distribution, while the average height and width distribution decreased for the TBA-salt polymers and increased for P75-$LiClO_4$ after exposure and electrochemical doping. Note that the height distribution size (X-axis) represents the relative size feature to each scan due to the lack of a common zero baseline.

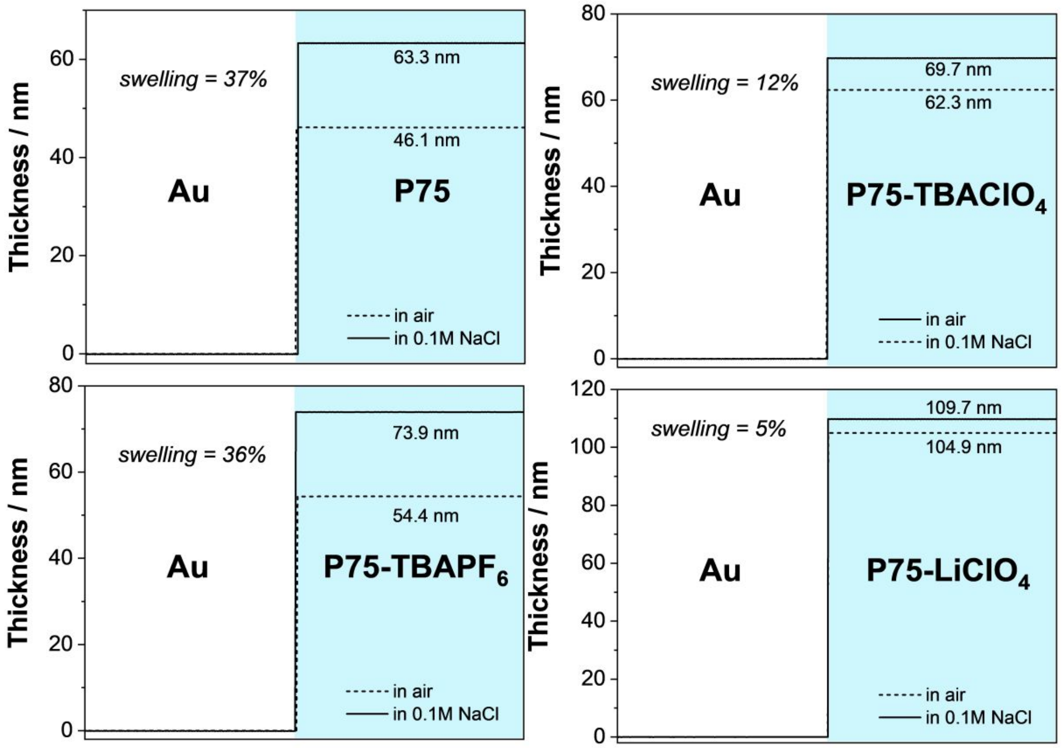

Figure S19. Mass changes in the films when immersed in 0.1 M NaCl. The thicknesses are shown for the dry (dashed lines) and swollen states (solid lines) of P75, P75-TBAClO_4, P75-TBAPF_6, and P75-LiClO_4 films.

Table S4. Swelling parameters of P75, P75-TBAClO_4, P75-TBAPF_6, and P75-LiClO_4.

Polymer	Passive swelling (%)	Active swelling (%) (*vs*. dry)	Active swelling (%) (*vs*. wet)	V_{OC} (V)
**P75**	37	47	7	-0.02
**P75-TBAClO_4**	12	18	5	-0.03
**P75-TBAPF_6**	36	44	6	-0.04
**P75-LiClO_4**	5	9	4	-0.02

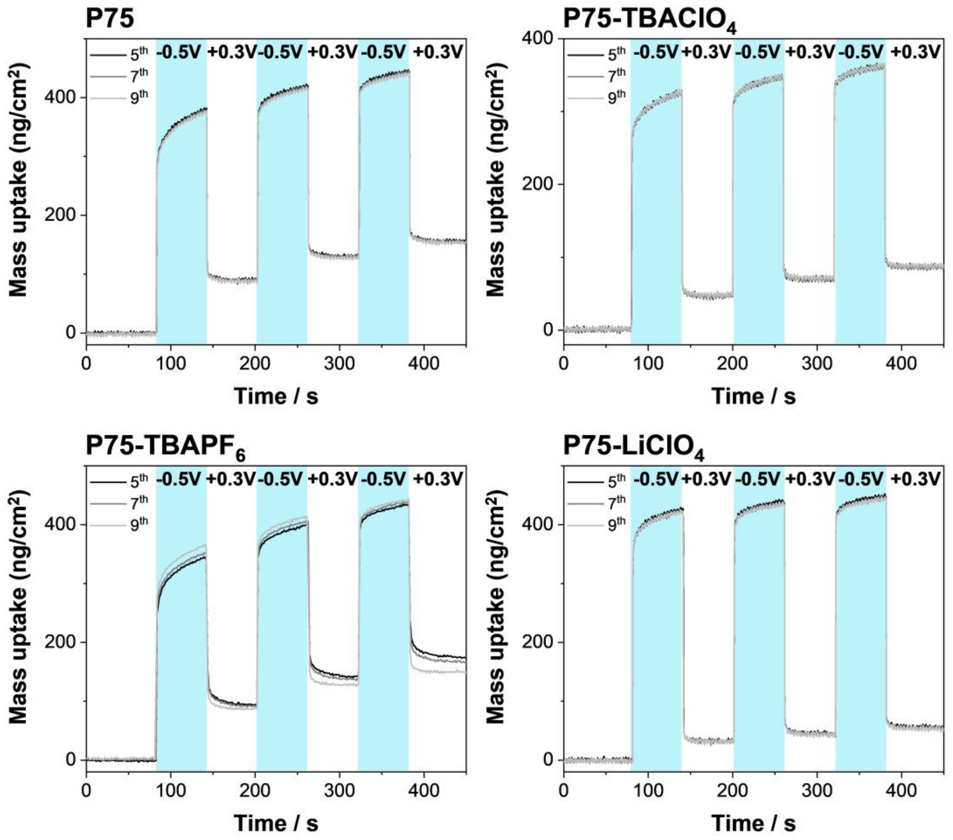

Figure S20. Mass changes of the films during electrochemical doping (-0.5 V) and dedoping (0.3 V) using Ag/AgCl in 0.1 M NaCl solution.

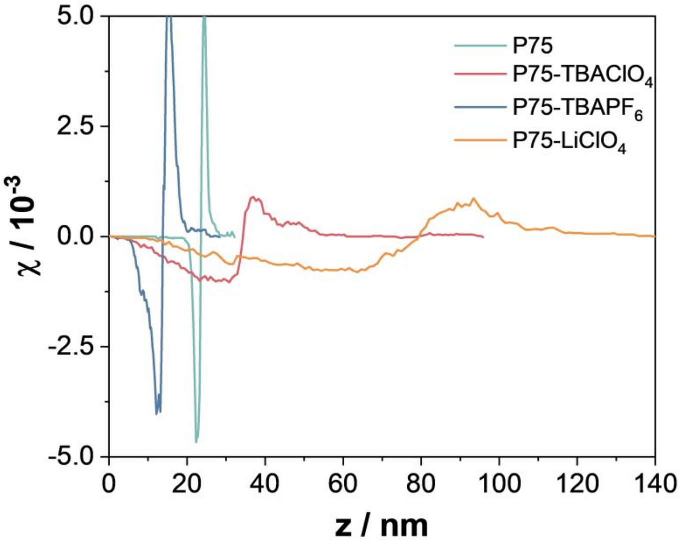

Figure S21. Minkowski connectivity function extracted from AFM "Dry after swelling" images.

Minkowski quantities offer an integrated approach from the integral geometry using height as the threshold variable to describe the sample surface characteristics (volume, boundary, and connectivity). **Figure S21** represents the character of the Minkowski connectivity function distribution. The Minkowski connectivity (χ) curves for all the polymers present an oscillatory form. Valleys are indicated by negative values where the minimum corresponds to the highest density of valleys. Conversely, positive values represent peaks, and the maximum is the highest density of peaks. P75-TBAClO_4 and P75-LiClO_4 Minkowski connectivity curves are both dominated by negative values, indicating the dominance of valleys (pores) over peaks on the surface. Alternatively, the pristine P75 shows a very sharp transition around 20 nm, characteristic of an overall smoother surface. Additionally, the oscillatory behavior of the Minkowski connectivity curves has previously been used to describe randomly and poorly connected valleys/peaks.[1] The lower strength of the oscillatory behavior for P75-TBAClO_4 and P75-LiClO_4 indicates a more interconnected pore network than for the other films. This is in line with the generally improved ion transport observed for these films (e-QCMD).

References

[1] F. M. Mwema, E. T. Akinlabi, O. P. Oladijo, *Sputtered Thin Films: Theory and Fractal Descriptions*, CRC Press, **202.**
